# Rodent home cage monitoring for preclinical safety pharmacology assessment: results of a multi-company validation evaluating nonclinical and clinical data from three compounds

**DOI:** 10.3389/ftox.2025.1655330

**Published:** 2025-11-05

**Authors:** R. R. Sillito, J. Sutherland, A. Milne, C. Giuliano, C. Sigfridsson, M. Rolf, A. K. Cherian, M. McClafferty, E. I. Rossman, G. Teuns, J. D. Armstrong, A. M. Holmes

**Affiliations:** ^1^ Actual Analytics Ltd., Edinburgh, United Kingdom; ^2^ Charles River Laboratories, Elphinstone Research Centre, Tranent, United Kingdom; ^3^ ICON Plc, Livingston, United Kingdom; ^4^ AstraZeneca, R&D Biopharmaceuticals, The Discovery Centre, Cambridge Biomedical Campus, Cambridge, United Kingdom; ^5^ AstraZeneca, R&D Biopharmaceuticals, Mölndal, Sweden; ^6^ GlaxoSmithKline, Collegeville, PA, United States; ^7^ Preclinical Sciences and Translational Safety, Janssen R&D, a Pharmaceutical company of Johnson & Johnson Innovative Medicines, Turnhoutseweg, Belgium; ^8^ School of Informatics, University of Edinburgh, Edinburgh, United Kingdom; ^9^ National Centre for the Replacement, Refinement and Reduction of Animals in Research, London, United Kingdom

**Keywords:** safety pharmacology, home cage monitoring, CNS effects, non-evoked behaviour, longitudinal behavioural assessment, preclinical animal models, functional observational battery, FOB/Irwin

## Abstract

**Introduction:**

The presence of central nervous system (CNS) safety concerns during early clinical testing that were not picked up in standard preclinical assessment is a major cause of attrition in drug development. It is also very expensive, time consuming and potentially dangerous for clinical trial participants. Rodent home cage monitoring approaches have previously been shown to deliver significant animal welfare benefits through group/social housing, minimal interventions avoiding stress that can confound results, and in some cases also animal reduction benefits with the multiplex data acquisition requiring fewer total animals. Here we looked at the utility of home cage monitoring to uncover potential CNS effects not identified using standard safety pharmacology tests.

**Method:**

We hypothesised that longitudinal behavioural assessment–by capturing non-evoked behaviour and reducing sampling artefacts–would be more sensitive to adverse reactions in preclinical animal models (i.e., rodents). To test this, we selected three compounds which previously passed standard safety tests but were failed later including two during clinical trials. We validated the general methodology for using home cage monitoring in safety assessment study designs from single doses to repeat dosing for up to 4 weeks. We then re-tested the three compounds in single dose studies.

**Results/Discussion:**

We showed that the methodology fits well with standard study designs. More importantly we uncovered significant findings in all three compounds that were not observed in the original classic safety pharmacology tests. The lack of such effects observed in standard preclinical assessment likely reflects functional differences between the limited observational snapshots characteristic of this approach and the more comprehensive temporal resolution enabled by continuous home cage monitoring.

## 1 Introduction

Central nervous system (CNS) safety pharmacology assessment is included within the ‘core battery’ assessment of vital organ functions as stated in the ICH S7A guidelines (ICHS7A, 2001). This traditionally relies on the Functional Observational Battery (FOB) or Irwin/modified Irwin test conducted in rodents (Baldrick, 2021; Jackson et al., 2019; Mathiasen and Moser, 2023; Redfern et al., 2019) although other methods are also acceptable. The parameters in the FOB and Irwin tests overlap and are to some extent interchangeable (Gauvin et al., 2016; Gauvin et al., 2019).

The FOB/Irwin is a subjective assessment of several different aspects of animal behaviour (ranging from general appearance and activity, though to innate reflexes and motor coordination) performed in a standardised manner by experienced observers (Redfern et al., 2019). Collection of these data for regulatory submission is usually performed as a ‘standalone’ safety pharmacology study, although increasingly, the standalone study is being replaced by observations performed within regulatory repeat-dose toxicology studies (Lindgren et al., 2008).

Adverse CNS findings remain one of the major contributors to delays and failure during drug development (Gribkoff and Kaczmarek, 2017; Mead et al., 2016; Valentin and Leishman, 2023). The low impact of the FOB/Irwin on progression of new chemical entities (NCEs) to human trials has been highlighted previously (Jackson et al., 2019; Mead et al., 2016), bringing into question the utility and validity of this test. As with many preclinical assessments (including the home cage monitoring approach proposed here), the FOB/Irwin parameters do not always have obvious clinical correlates (Mead et al., 2016; Tamaki et al., 2013), and their translation to human outcomes is confounded by fundamental differences between the behavioural domains of rats and humans. Beyond this common limitation, however, a key challenge with the FOB/Irwin is that it entails a subjective assessment over a large number of recorded parameters. Furthermore, the measurement of FOB/Irwin parameters is inherently episodic, leaving animals unobserved for the majority of the study period, most notably during the dark phase when rodents are most active. A fundamental property of the FOB/Irwin is that every measured parameter requires the presence of a human observer, and many require handling and removal from the home cage: this approach gives very little scope for measurement of non-evoked behaviours and may thus limit observation of drug effects (Gouveia and Hurst, 2017).

**FIGURE 1:**
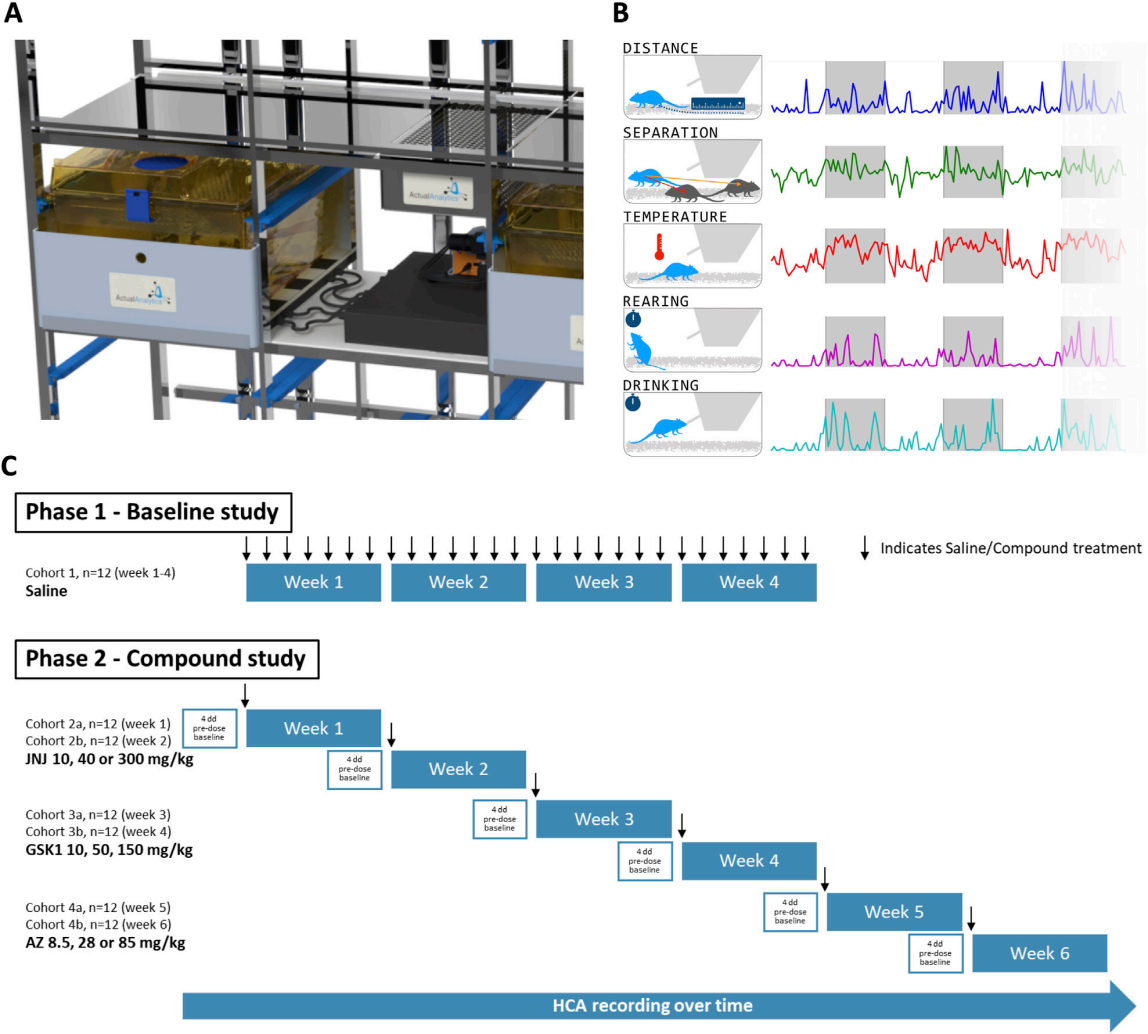
Overview of Home Cage Analyser (ActualHCA) and study design. **(A)** ActualHCA monitors rats 24/7 in regular home cages using a combination of IR video from the right-hand side with RFID telemetry on a 2D matrix underneath the cage. **(B)** Parameters detected by the ActualHCA system in this study. **(C)** Schematic of the experimental design and dosing schedule. In Phase 1, 12 rats were given a vehicle dose daily, and monitored over 28 days to validate the system under the conditions of a repeat-dose toxicology study. In Phase 2, rats were monitored over 11 days–with days 2–4 providing baseline measurements, prior to dosing on day 5 (with either vehicle or one of three compound dose levels); monitoring then continued for a further 5 days. This protocol was repeated twice for each compound (JNJ, GSK, and AZ) comprising a total of 6 cohorts of 12 rats–see text for details.

Home cage analysis systems provide an opportunity to monitor the non-evoked behaviour of animals within social groups (for review of available home cage monitoring technologies see Klein et al. (2022)). In the present study we used the ActualHCA system (HCA, Actual Analytics Ltd. UK), which supports continuous collection of rodent temperature and behavioural data, including ambulatory and vertical activity, drinking and interaction between cage mates (see Methods and Figure 1). All recordings are performed in the home cage, with no requirement to handle or remove animals from the cage^1^. Importantly, monitoring is continuous, allowing animals to be observed for entire periods following dosing, rather than at selected timepoints, and also during the dark phase, when rodents are more active and effects on behaviour may still be present.

Tse et al. (2018) previously demonstrated that the HCA could detect effects on ambulatory and vertical activities, and on subcutaneous temperature for the test agents chlorpromazine, clonidine and amphetamine. The observed findings were consistent with the modified Irwin test (Redfern et al., 2019) and the known pharmacology of these test agents in rats.

All previous home cage studies have used compounds where effects would be expected to be observed in standard test batteries. We hypothesised that the HCA should be able to detect behavioural changes indicative of possible CNS effects that would be missed using standard methods. To test this, we selected three historical compounds which had been evaluated in the modified Irwin test as part of the ‘core battery’ safety pharmacology assessment according to ICH S7A. Two of these compounds entered phase I clinical trials but were not progressed further due to CNS findings. Development of the third compound was terminated because of serious adverse CNS and systemic effects seen in other preclinical testing (please see Discussion for details of these previous CNS findings).

The present study comprised two separate experimental phases. Phase 1 was a baseline study where we first evaluated the utility of HCA under similar procedures and conditions as those employed during regulatory toxicology studies where animals were dosed daily for 28 days. In this phase, animals were dosed solely with vehicle and were continuously monitored using the HCA system. The results confirmed that home cage monitoring was technically compatible with typical single and repeat dose study designs. In Phase 2, three historical candidate drugs were tested and the results and decision-making potential of HCA compared versus the modified Irwin screen. This latter phase showed that the HCA can detect effects of drugs not seen in the modified Irwin which could inform future development.

## 2 Materials and methods

### 2.1 Animals and husbandry

All animal experiments were conducted in accordance with animal welfare guidelines of Charles River, Edinburgh and licensed by UK Home Office under the Animals (Scientific Procedures) Act 1986 (Licence no PP7731880, Protocol 2). The regulations conform to EU Directive 2010/63/EU and achieve the standard of care required by the US Department of Health and Human Services’ Guide for the Care and Use of Laboratory Animals.

Male rats (Wistar Han Crl:WI(Han) 9–12 weeks), weighing between 262 g and 447 g on the days of dosing, were socially housed (three per cage) throughout the experiments in industry standard, appropriately sized polycarbonate IVC cages with stainless steel grid tops and solid bottoms (1500U, Tecniplast S. p.A, Italy). Sterilised white wood shavings were used as the bedding material (Datesand Ltd., UK.). Animals were provided with devices for hiding in (e.g., PVC pipe, Datesand Ltd., UK.) and objects for chewing (e.g., aspen wooden blocks, Tapvei^®^) for psychological/environmental enrichment. The facility provided a temperature- (19–23 °C) and humidity-controlled (40%–70%) environment on a 12/12 h light-dark cycle (lights on between 7a.m. and 7p.m.) with food (SDS Rat and Mouse (modified) No. 1 Diet SQC Expanded, Special Diet Services Ltd. Germany) and water available *ad libitum*. Animals were allowed to acclimatise to the facility for a period of 10 days before commencement of dosing.

### 2.2 Procedure for implantation of RFID chips

Each animal was identified with a subcutaneously implanted temperature sensitive passive radiofrequency identification (RFID) transponder (BioTherm13, Biomark, USA).

Animals were anaesthetised for implantation using isoflurane (3%–5% with oxygen at 2 L/min). The transponders were implanted subcutaneously into the ventral abdomen using a pre-sterilised trocar needle, pre-loaded with the transponder. Following implantation, animals were checked at least twice daily and allowed to recover for at least 5 days (Redfern et al., 2017).

### 2.3 Experimental design

Throughout this study the experimental unit was the individual rat, monitored in a group-housed environment. The full study consisted of two separate experimental phases (Figure 1C): i) Phase 1 – Baseline study; ii) Phase 2 – Compound study. In Phase 1, rats (n = 12, four cages of three animals) were dosed daily with 0.9% saline for 28 days under similar procedures and conditions employed during regulatory toxicology studies (Supplementary Figure 1). In Phase 2, each dose of each of the three drugs was evaluated in two runs of three animals each (n = 6 per dose group per compound) for 4 days pre-dose, on the day of dosing and up to 5 days post-dose (Figure 1C). Within each cage, all animals received the same dose level. The total number of rats used in Phase 2 was 72. Each treatment was administered as a single dose by oral gavage in a dose volume of 5–10 mL/kg. The experimental runs for a treatment were conducted in consecutive weeks. No inclusion or exclusion criteria were applied to any animals during the experiments.

### 2.4 Validation of sample size

The primary justification for the number of animals was to replicate the conditions of the modified Irwin test (Ewart et al., 2013). Whilst the proposed sample size has been established as suitable for the Irwin test, we have also demonstrated its appropriateness for the compound phase of the present study on the basis of previous validations of HCA in a pharmacological context. Notably, the same sample size (n = 6 per dose group) was sufficient to reveal significant effects in a previous study using several different compounds (Tse et al., 2018), where a single dose level was compared to vehicle. Applying this approach here, 24 animals split equally between 4 treatment groups (vehicle, low, medium and high doses), is sufficient to detect an effect size of f = 0.74 (assuming α = 0.05, 80% power, and a one-way ANOVA test) (Faul et al., 2007). This is commensurate with locomotor effect sizes previously observed for compounds tested with HCA, where deviation of treated animals (expressed here as Cohen’s f) from the vehicle group ranged through 0.56 (PCP (Mitchell et al., 2020)), 0.76 (Amphetamine (Tse et al., 2018)), 0.81 (Clonidine (Tse et al., 2018)) and 0.84 (Chlorpromazine (Tse et al., 2018)), suggesting that the proposed sample size would be adequate to reveal effects for the three further compounds considered in this paper.

### 2.5 Home cage monitoring system

The HCA (Actual Analytics UK) system has been described in detail elsewhere (see Redfern et al. (2017)). In brief, each animal carried a unique RFID identity tag which also reports subcutaneous temperature. The home cage rests on a 3 × 4 array of RFID antennae that scan the entire floor reporting animal identification, spatial location and temperature at roughly 1 Hz. The cage is also illuminated by an array of infrared LED lights and a side mounted camera captures a 24/7 video record of the events within the cage. These raw data sources are combined and analysed to generate a longitudinal behavioural profile for each individual animal consisting of several parameters, including distance moved, time spent rearing (vertical motion), temperature, time spent drinking and social separation. Each of these parameters can then be examined over multiple timescales and time bins of interest.

### 2.6 Compounds for administration and dose selection

Three compounds (JNJ, GSK and AZ) were selected for testing in the compound study phase of the study (Phase 2 – Compound study). These compounds had previously been assessed via oral gavage in the modified Irwin test with compound-related findings that were not considered an impediment to further development. For this phase of the study, dose levels for all three compounds were selected based on the previous tests as follows:

JNJ: The compound is a high affinity antagonist of human adenosine receptors which was selected as a drug candidate for the treatment of Parkinson’s disease. JNJ was assessed in the modified Irwin test in two strains of rat; male Sprague Dawley rats at single doses of 10, 40 or 300 mg/kg body weight and male Wistar Han rats at 300 mg/kg. At this high dose, an increase in locomotor activity up to 7 h post-dosing (Sprague-Dawley) or 24 h (Wistar Han), a higher incidence of response to touch escape, a slightly larger pupil size, and a decreased defecation rate were noted in both strains. The lower doses tested in Sprague-Dawley rats only led to increased locomotor activity and increased body temperature. The low dose level, 10 mg/kg, was expected to achieve maximum plasma concentration approximately 80-fold higher than the clinically efficacious concentration. Based upon these results, single doses of 10, 40 and 300 mg/kg were selected for assessment in the compound study phase.

GSK: The compound is a 4-aminoquinoline antimalarial that demonstrates low to moderate clearance and excellent oral bioavailability with linear pharmacokinetics. In previous studies, single oral doses of GSK at 10 or 50 mg/kg were tolerated with no adverse effects on neurobehavioral, body temperature or clinical observations in male Sprague-Dawley (SD) rats. In repeat dose studies in rats, CNS effects were evident after four daily doses of ≥150 mg/kg/day and presented as underactivity, partially closed eyelids, hunched posture, abnormal gait and piloerection. Due to the increasing severity of these clinical signs, the study was terminated on day seven. In addition, body weight loss was observed at ≥150 mg/kg/day while body weight gain was reduced for animals given 10 or 50 mg/kg/day. Based on these findings, the doses of GSK selected for the compound study phase were 10, 50 and 150 mg/kg.

AZ: The compound is a negative allosteric modulator of the metabotropic glutamate receptor subtype 5, developed for multiple neuroscience and gastrointestinal indications. Single oral doses of AZ nanosuspension at 8.5, 28 or 85 mg/kg were selected based on results from previous studies in the rat (Wistar Han, male). The high dose was well tolerated in several rat studies. The low dose level, 8.5 mg/kg, was expected to achieve maximum free plasma concentration approximately 5 to 10-fold higher than the clinically efficacious concentration. Transient effects were observed in several behavioural measures at all doses up to 60 min after dosing. No effect was detected 60 min or thereafter at any dose.

For all compounds, welfare monitoring consisted of cage-side observations where each animal was checked for its general wellbeing. This was conducted immediately post-dose and at least twice daily for the duration of the experiment. No adverse effects were noted and these observations do not form any part of the reported data.

### 2.7 Formulation

The vehicle for JNJ was 20% (w/v) hydroxypropyl-β-cyclodextrin. Vehicle for GSK was 1% (w/v) aqueous methylcellulose and for AZ was 1.0% (w/w) polyvinylpyrrolidone and 0.2% (w/w) disodium salt in deionised, distilled water stabilised nanosuspension. Dosing formulations were stirred continuously for at least 30 min prior to dosing and throughout the dosing procedure.

### 2.8 Bioanalysis

A single blood sample was taken from all animals at 2 h post-dose (JNJ), 4 h post-dose (GSK) or 24 h post-dose (AZ), to confirm exposure. Blood collection from animals receiving JNJ and AZ was by microsampling (32 µL) from the tail vein. A 0.2 mL sample was taken from the jugular vein from animals receiving GSK. In line with previous investigations of the three compounds, all blood sampling took place without anaesthesia.

Samples were analysed by LC-MS/MS using qualified research methods (Adaway and Keevil, 2012; Korfmacher, 2005; Muck, 1999).

### 2.9 Data analysis

The continuous video and RFID data streams captured by the HCA systems provide multiple parameters which can be analysed over a wide range of temporal granularities. Our approach for analysing this data in the present study is described in the sections below.

#### 2.9.1 Blinding

Particular emphasis was placed on blinding in the study such that the data analytics team (Authors JDA and RS) was excluded from compound selection discussions and kept completely blind to compound names, the class of molecules included in the study and all previous findings/observations with those compounds until after the data were collected, processed and analysed as described below. After the authors were finally briefed, an additional time window was added to the analysis, to allow comparison with previous data collected for the AZ compound.

#### 2.9.2 Measurements

For each compound, we measured horizontal locomotion, rearing behaviour (vertical locomotion), subcutaneous body temperature, social separation (distance to nearest other animal) and drinking from the waterspout (see Figure 1). We focussed in particular on horizontal locomotion, rearing, and body temperature, given their prior validation and overlap with the modified Irwin test (Tse et al., 2018). Longitudinal profiles for all of these measures in each of the test and control groups are provided in Supplementary material (graphs and summary data in 10- and 60-min bins), but for the purposes of analysis we considered these profiles in terms of temporal averages calculated for a specific set of time windows of interest.

#### 2.9.3 Selection of time windows

In the absence of information about the compounds, our hypothesis was simply that there would be some deviation from the control group occurring over an unknown timescale from dosing onwards. Our approach was to consider a broad selection of time windows. With the exception of the first hour after dosing, we considered the timeline in terms of 3 h “super-intervals”, and aggregations thereof, from the time of dosing onwards.

Light phase (on dosing day):

• 0–1 h after dosing (revealed to be salient in previous data from AZ compound).
• 0–3 h after dosing.
• 0–9 h after dosing, (until lights-off).
• 3–6 h after dosing (encompassing blood sampling time for GSK).

Dark phase (on dosing day):

• 0–3 h after lights-off.
• 0–12 h after lights-off (until lights-on).

For some compounds, we also considered additional time windows, driven by the observed duration of effects visible in the temporal traces plotted for dosing day:

• Light phase: 3–9 h after dosing.
• Dark phase: 3–12 h after lights-off, (until lights-on).

Finally, driven by initial multi-day exploratory plots of the data, we also considered the possibility of longer-term effects over the days after dosing day:

• Average dark phase (0–12 h after lights-off) over the 5 days after dosing day.

For each of these time windows, we calculated a temporal average for every parameter of interest, before applying a baseline-correction as described below.

#### 2.9.4 Baseline correction

While the vehicle treated group provides the principal comparator for assessing compound effects, these effects can only be observed against the backdrop of the natural variation between cage groups of animals where, for example, one group may already be intrinsically slightly more or less active than another. The longitudinal nature of the HCA recordings provides the opportunity to mitigate this effect, by capturing the animals’ baseline behaviour prior to dosing.

For all recordings, we used the three full days before dosing to obtain (for every measurement) a “baseline” 24 h temporal profile for each cage group. This allowed us to calculate a baseline value for each time window/measurement of interest (for example, average temperature between 10a.m. and 1p.m.), which was then subtracted from the corresponding value measured after dosing, yielding a set of “difference from cage-group baseline” values for the six animals within each treatment group.

#### 2.9.5 Statistical analysis

For each time window and measurement of interest, the presence of a statistically significant (p < 0.05) difference between baseline-corrected values for the four treatment groups (vehicle, low, medium and high-dose) was assessed using a one-way ANOVA test. Wherever a significant difference was found between these groups, Dunnett’s test was then used to establish which of the dose levels yielded a statistically significant (p < 0.05) deviation from the vehicle group.

Given the exploratory nature of the blinded analysis, we were mindful of the possibility of generating Type I (false positive) errors as a result of the large number of time windows under consideration on dosing day. We therefore applied the Benjamini Hochberg False Discovery Rate (FDR) control procedure to provide a more stringent filter on the one-way ANOVA p-values obtained within each compound, allowing us to enumerate a superset of all time window/parameter combinations, and identify those which met the 5% FDR control level, under the unfavourable assumption that every possible combination had been considered.

## 3 Results

### 3.1 Establishing baselines (phase 1 – baseline study)

Prior to performing the compound study phase (Phase 2 – Compound study), we investigated in a baseline study phase (Phase 1 – Baseline study) how variable the behaviour of the animals would be in a safety/toxicology environment, with daily handling for dosing. A typical GLP toxicity study supporting first-in-human (FIH) studies is of 4 weeks duration, so we designed a simple non-GLP study with 12 control animals housed in the HCA systems and monitored for 28 days (Supplementary Figure 1). Each day the animals were administered saline by oral gavage. There was a clear behavioural response with increased locomotor activity in the hour immediately after the dosing event after which activity levels subsided to the normal low level daytime activity. Activity increased as expected during the night phase. There was a modest habituation to the dosing procedure which could be seen as a progressively faster recovery time post-gavage as the weeks progressed. The activity profiles also increased slightly in response to technicians entering the room for routine checks and during cage changes. In summary all operator interventions (dosing, routine checks and cage changes) induced a short increase in locomotor activity which was overlaid onto the otherwise robust day/night cycle of activity.

### 3.2 Key findings by compound (phase 2 – compound study)

The key significant compound-induced changes in behaviour are described below, with the full set of behavioural changes for the 24 h following dosing enumerated in Table 1. For a detailed description of how the data was processed and these behavioural changes determined please refer to the ‘Data analysis’ section in the materials and methods.

**TABLE 1:** Dosing day results for all three compounds Baseline-corrected measurements are shown here for all three compounds JNJ, GSK and AZ. In each case all five measurements captured by the HCA system are shown, across a common “super-set” of time windows–encompassing all time windows considered in our initial blinded exploratory analyses. For each parameter/time-window, the average (n = 6) baseline-adjusted measurements are shown for each dose level: for all non-zero dose levels these entries are coloured by their deviation from the Vehicle measurements (calculated using Glass’s Delta, i.e., using the mean and variance of the Vehicle group). Combinations of parameter and time-window are highlighted with asterisks wherever the omnibus test (One-Way ANOVA) showed a significant (*p < 0.05, **p < 0.01, ***p < 0.001) difference between the four dose levels. Results of the omnibus test are only shown wherever the calculated p-value meets the criterion for either 5% (boxes with dashed lines) or 10% (boxes with dotted lines) False Discovery Rate correction following the Benjamini-Hochberg procedure (conducted separately for each compound). We adopted the 5% threshold as a precondition of any results declared as significant on dosing day, given the large number of time-windows under consideration.

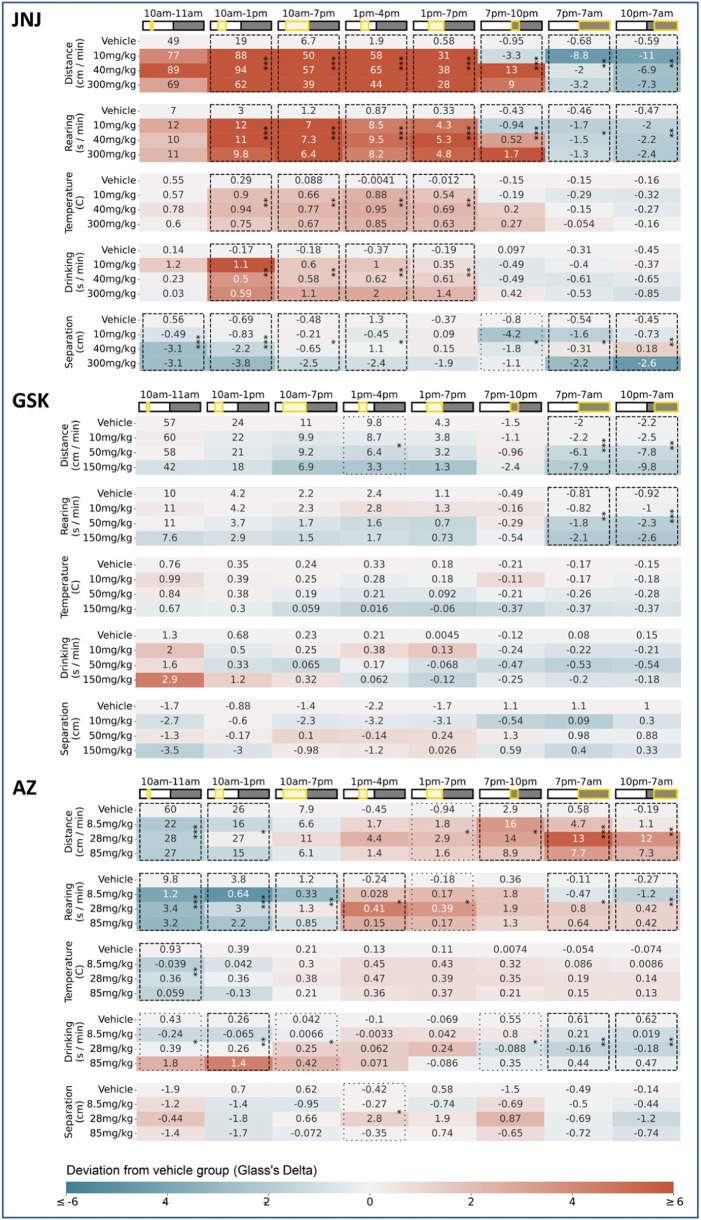

**FIGURE 2:**
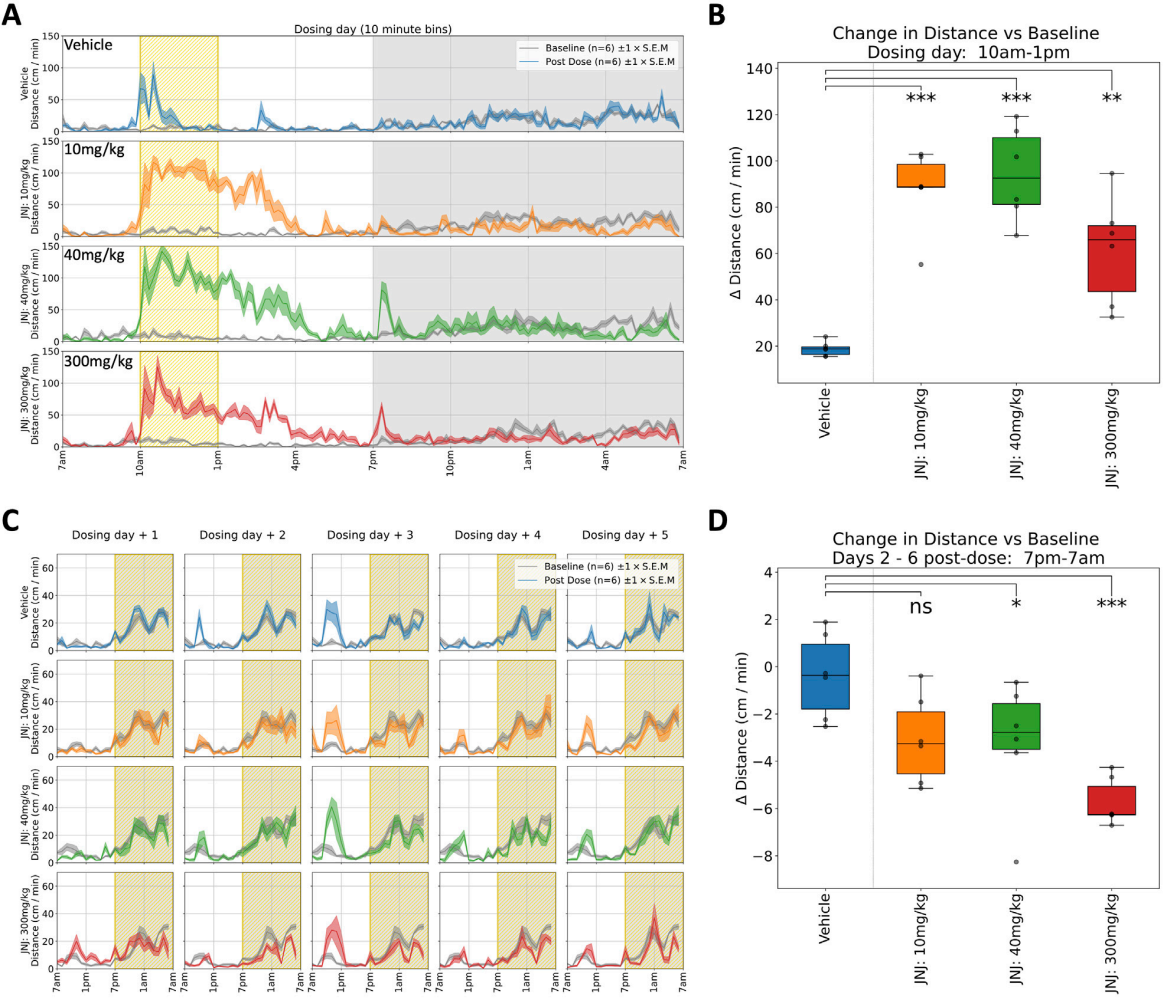
Selected responses to a single dose of JNJ. **(A)** Locomotor activity (distance travelled per minute) across 24 h on dosing day. Mean activity (±S.E.M.) is shown for each treatment group (N = 6), alongside an additional gray trace representing baseline activity (averaged over the preceding days) for the same group over an equivalent 24 h time period. A sustained period of hyperactivity can be seen starting at 10a.m. around dosing. Conversely, activity is suppressed especially in the period from 10p.m. through to 7a.m. **(B)** Locomotor activity was significantly increased (relative to baseline) between 10a.m. and 1p.m. for all doses: 10(***), 40(***) and 300 mg/kg (**). † **(C)** Locomotor activity traces for 5 days following dosing day. (Please note that the same baseline trace, derived from days 2–4, is repeated within each dose level.) **(D)** Dose dependent decrease (relative to baseline) in average night-time locomotor activity across the 5 days, with significant decrease at 40 (*) and 300 mg/kg (***) doses†. †Asterisks denote significant (*p < 0.05, **p < 0.01, ***p < 0.001) differences to Vehicle group according to Dunnett’s test, conducted wherever a one-way ANOVA test showed a significant (p < 0.05) effect of treatment across the four groups. Box plots show the center line at the median, with whiskers at the furthest data points within ±1.5 x interquartile range, and raw data (n = 6) overlaid as grey circles. On Dosing Day (where multiple time-windows were considered), ANOVA results were not considered significant unless they also met a Benjamini-Hochberg False Discovery Rate threshold of 5%, applied across all possible permutations of parameter and time period for any given compound (please refer to Table 1 for full enumeration of dosing-day results).

JNJ: Rats received a single oral dose of either vehicle or JNJ (10, 40 or 300 mg/kg) and were monitored for 6 days. In the first hours following administration (during the light phase), the JNJ compound, at all doses, increased horizontal locomotor activity (Figures 2A,B) and rearing, which coincided with a slight increase in body temperature. The hyperactivity and increased body temperature for the three doses are in line with previously reported modified Irwin test data. Following lights-off at 7p.m. there is an apparent dose-dependent increase in activity (see Table 1, 7p.m.–10p.m.), suggesting an evoked response that increases with dose level. However, taking the first dark phase after dosing in its entirety, we observed decreased horizontal locomotor activity and rearing, again across all doses (see Table 1 for details). Beyond dosing day, we also recorded decreased horizontal locomotor activity persisting over the subsequent five dark phases at 40 and 300 mg/kg with the strongest effect at 300 mg/kg (Figures 2C,D). Rearing also decreased at these doses and during the same timespan (data not shown). The latter observations are novel as previous modified Irwin tests did not include observations during the dark phase (see also Figure 5A). It is salient to note that T_max_ for JNJ occurs within 2 h of dosing, yet the effects of this single dose can be observed days later in the home cage.

**FIGURE 3:**
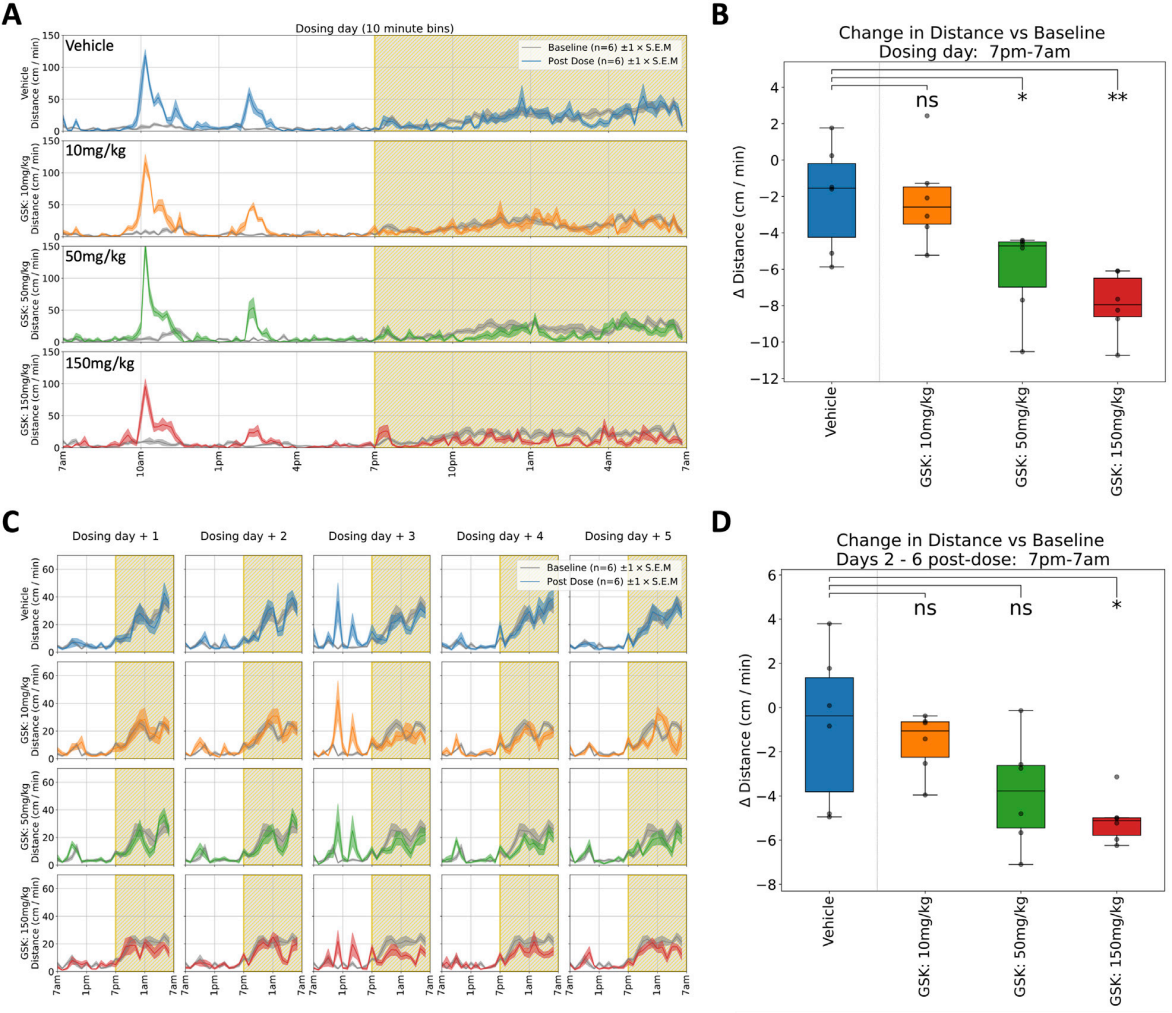
Selected responses to a single dose of GSK. **(A)** Locomotor activity (distance travelled per minute) across 24 h on dosing day. Mean activity (±S.E.M.) is shown for each treatment group (N = 6), alongside an additional gray trace representing baseline activity (averaged over the preceding days) for the same group over an equivalent 24 h time period. A brief spike in activity can be seen at 10a.m. around dosing time followed by a further increase around 2p.m. at blood sampling time. **(B)** Locomotor activity was significantly suppressed (relative to baseline) between 7p.m. and 7a.m. at 50 (*) and 150 mg/kg (**) doses †. **(C)** Locomotor activity traces for 5 days following dosing day. (Please note that the same baseline trace, derived from days 2–4, is repeated within each dose level.) **(D)** Dose dependent decrease (relative to baseline) in average night-time locomotor activity across the 5 days, with a significant decrease at highest 150 mg/kg (*) dose†. †Asterisks denote significant (*p < 0.05, **p < 0.01, ***p < 0.001) differences to Vehicle group according to Dunnett’s test, conducted wherever a one-way ANOVA test showed a significant (p < 0.05) effect of treatment across the four groups. Box plots show the center line at the median, with whiskers at the furthest data points within ±1.5 x interquartile range, and raw data (n = 6) overlaid as grey circles. On Dosing Day (where multiple time-windows were considered), ANOVA results were not considered significant unless they also met a Benjamini-Hochberg False Discovery Rate threshold of 5%, applied across all possible permutations of parameter and time period for any given compound (please refer to Table 1 for full enumeration of dosing-day results).

GSK: Rats received a single oral dose of either vehicle or GSK (10, 50, or 150 mg/kg) and were monitored for 6 days. A dose-dependent reduction in horizontal locomotor activity (Figures 3A,B) and rearing was observed in both the 50 and 150 mg/kg groups during the first dark phase following dosing (see Table 1 for details). There is also a (non-significant) dose-dependent decrease in the *evoked* activity surrounding the blood sampling event at 4 h post dose (Figures 5D,E.) It is notable that the suppression of locomotor activity persisted, in a dose dependent manner, for five more days post-dose (Figures 3C,D). As with the preceding compound, T_max_ occurs within 8 h of dosing, which is at odds with the long-term effect observed here in the home cage. These observations diverge from previous modified Irwin tests where 50 mg/kg was well tolerated with no adverse effects on neurobehaviour, although these did not include observations during the dark phase (Figure 5B) when hypoactivity was reported in the current study.

**FIGURE 4:**
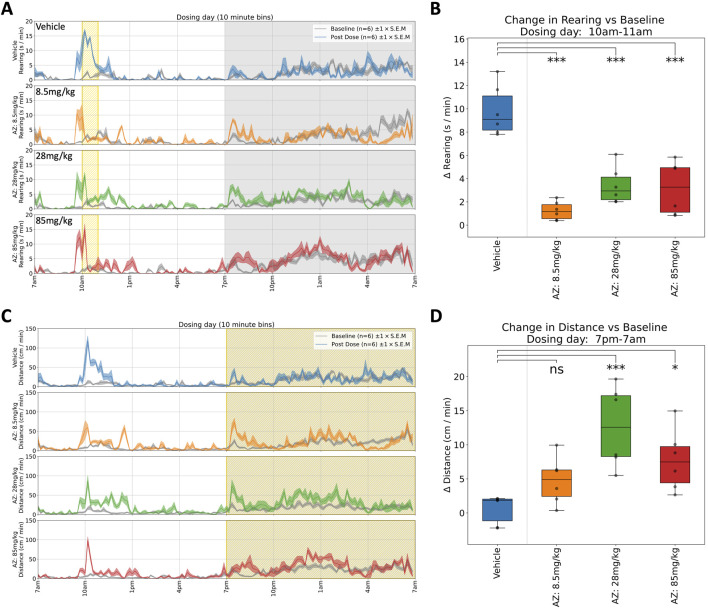
Selected responses to a single dose of AZ. **(A)** Rearing activity (time spent rearing per minute) on dosing day. Mean activity (±S.E.M.) is shown for each treatment group (N = 6), alongside an additional gray trace representing baseline activity (averaged over the preceding days) for the same group over an equivalent 24 h time period. All animals responded to dosing with an initial increase in rearing activity, but those treated with AZ showed a reduction in rearing activity compared to the control group (which remained hyperactive, relative to baseline, for over 1 h). **(B)** Compared to Vehicle-treated animals, rearing activity was significantly decreased (relative to baseline) between 10a.m. and 11a.m. for all doses: 8.5 (***), 28 (***) and 85 mg/kg (***).† **(C)** Locomotor activity traces (distance travelled per minute) on dosing day. Initial responses after dosing are similar to those shown for rearing, but the locomotor activity measure also reveals a pronounced increase in activity during the night-time. **(D)** Locomotor activity was significantly increased (relative to baseline) between 7p.m. and 7a.m. for the 28 (***) and 85 mg/kg (*) doses†. †Asterisks denote significant (*p < 0.05, **p < 0.01, ***p < 0.001) differences to Vehicle group according to Dunnett’s test, conducted wherever a one-way ANOVA test showed a significant (p < 0.05) effect of treatment across the four groups. Box plots show the center line at the median, with whiskers at the furthest data points within ±1.5 x interquartile range, and raw data (n = 6) overlaid as grey circles. On Dosing Day (where multiple time-windows were considered), ANOVA results were not considered significant unless they also met a Benjamini-Hochberg False Discovery Rate threshold of 5%, applied across all possible permutations of parameter and time period for any given compound (please refer to Table 1 for full enumeration of dosing-day results).

**FIGURE 5:**
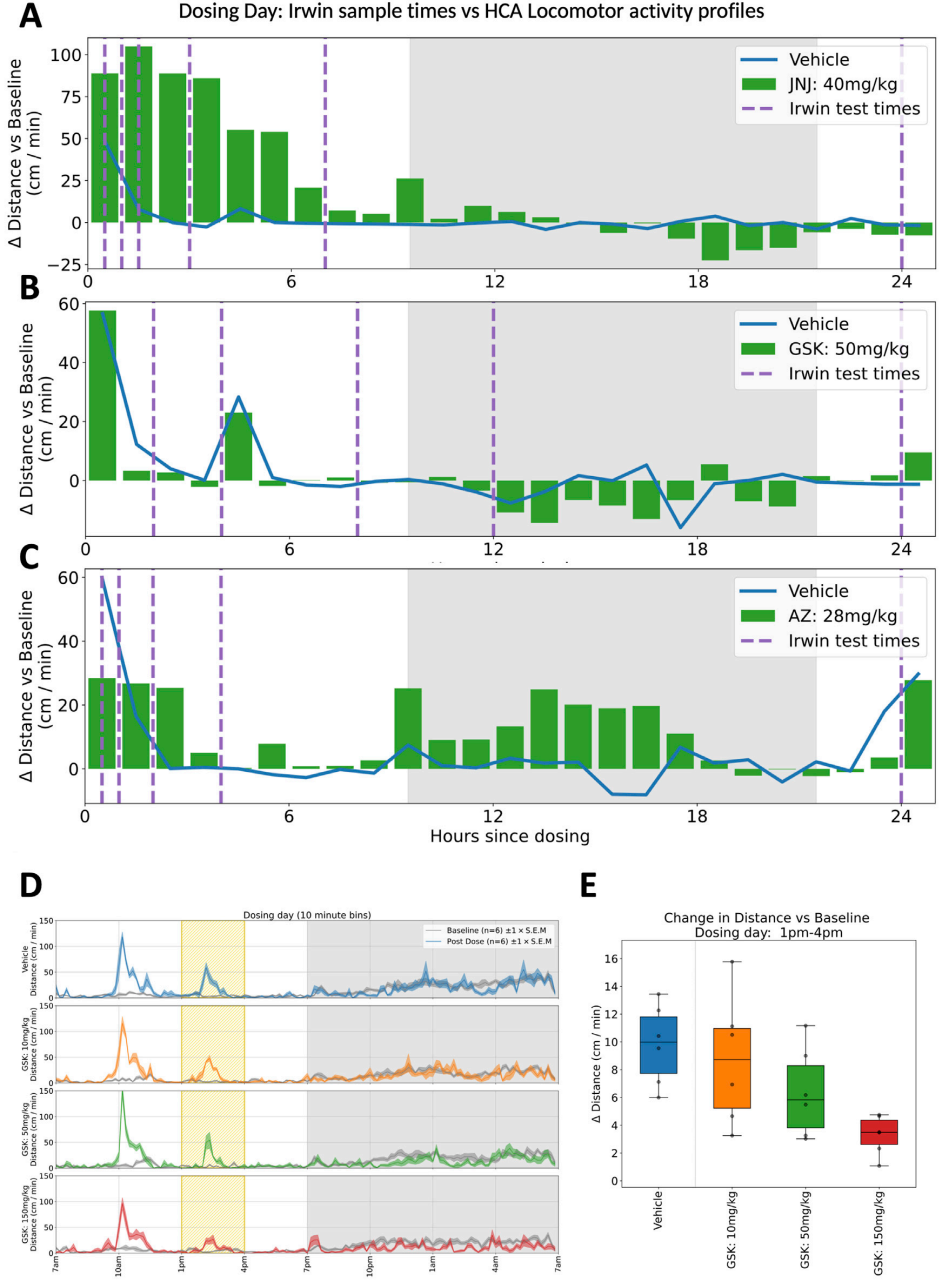
Comparing Irwin/FOB with Home Cage study designs. **(A–C)** Plots showing hour-by-hour deviations in locomotor activity (distance travelled per minute) relative to equivalent baseline average measurements, for 24 h following dosing time. **(A–C)** show responses to the mid-dose of compounds JNJ, GSK and AZ respectively. Sampling for the standard modified Irwin test are shown for each compound with dashed vertical lines. For the AZ compound shown in **(C)**, it is notable that some of the most pronounced deviation (in this case, hyperactivity), occurs during the 20 h between the final two sampling times. **(D)** Locomotor activity traces for compound GSK on dosing day, highlighting responses to a blood sampling procedure at 2p.m. This shows how a routine intervention can be framed as a challenge response within the context of home cage monitoring–crucially, the increase in activity that is typically recorded after handling interventions may be modulated by compound effects (Tse et al., 2018). **(E)** In the 3h window surrounding blood sampling (1p.m.–4p.m.), there is a dose dependent decrease in evoked activity for increasing concentrations of compound GSK, highlighting the value of home cage monitoring in capturing evoked responses in addition to non-evoked measurements.

AZ: Rats received a single oral dose of either vehicle or AZ (8.5, 28 or 85 mg/kg) and were monitored for 6 days. Dosing with AZ reduced both horizontal locomotor activity (Figure 4C) and rearing (Figure 4B) 1 h post-dose, at each dose. This effect was in line with the modified Irwin test on the compound. We observed increased body temperature 3–6 h post-dose but this effect was limited to a general trend and was not significant (see Table 1 for details). In the dark phase, we observed a significant increase in horizontal locomotor activity at both 28 and 85 mg/kg (Figure 4D). The latter observations were not reported in the original modified Irwin tests as these did not include assessment during the dark phase (Figure 5C).

### 3.3 Bioanalysis

Blood samples from each treatment group were processed for analysis of total plasma concentrations. Samples were analysed by LC-MS/MS using qualified research methods. The exposure to each compound was within the expected range for the dose levels administered, and comparable to previous exposure data obtained in the Irwin studies at the same dose levels. (Supplementary Figure 2).

## 4 Discussion

### 4.1 Establishment of baselines (phase 1 – baseline study)

The baseline study phase with saline dosing demonstrated that introducing HCA into the operational environment was not disruptive to the staff and standard operating procedures used. Further, it provided useful information on the sensitivity of the HCA to detect the impact of environmental changes and operator interventions, the animal-animal variation of such effects and their consistency over time. The absence of unusual or unexplained occurrences provides confidence in the ability of the HCA to reliably detect the effects of pharmacological interventions on behaviour.

### 4.2 Compound effects (phase 2 – compound study)

Dosing with all three compounds induced significant, measurable changes in non-evoked behaviour in the home cage when compared to vehicle controls and to the animals’ own pre-dose baseline recordings.

GSK: In the clinic, a seizure was observed in one subject following a single dose of GSK, stopping progression of the compound’s development. CNS effects had been reported at a high 150 mg/kg dose in a previous repeat dose investigative toxicology study, but no CNS effects were observed in the definitive GLP general toxicity or Irwin studies up to 50 mg/kg. In the present study using the home cage we clearly see behavioural effects at the lower, more clinically relevant dose. In addition, we see these behavioural effects at the higher dose of 150 mg/kg persisting for many days indicating a most unexpected suppression of normal dark phase activity in these animals which would probably have triggered more definitive studies (Figures 3, 5B).

JNJ: A similar story was seen with JNJ where an initial acute response was observed during the light phase which was in line with the report from the previous modified Irwin test results. However, dark-phase behaviour was not captured in the Irwin tests and therefore both short and long-lasting impacts on dark phase behaviour at the higher doses again reveals the increased sensitivity of the longitudinal data gathered in the home cage (Figures 2, 5A).

AZ: In line with the results from the other compounds, testing the AZ compound provided additional information compared to the modified Irwin assessment. Clinical development was discontinued due to lack of analgesic effects and dose-limiting anxiety and hallucination. Previous modified Irwin tests had indicated a suppression of locomotor behaviour that would be expected at the doses investigated and this was indeed also seen in the HCA system. The hyperactivity observed in the home cage in the first dark phase following dosing was not expected. While the effect was statistically significant, it is unlikely that this would have changed the benefit:risk assessment for the molecule in preclinical development. Instead, it is possible that additional assessments may have been incorporated into repeated dose toxicology studies to further characterise and investigate the effect (Figures 4, 5C).

In summary, three compounds which were previously assessed in the modified Irwin test as part of ‘core battery’ safety pharmacology assessment according to ICH S7A were tested using a home cage monitoring system. The longitudinal analysis made possible by this system revealed previously unreported behavioural changes associated with dosing with all three compounds. It is not clear if the new behavioural responses for AZ would have led to a negative outcome conclusion or additional evaluations. However, the dose-dependent and multi-day suppression of normal dark phase activity observed with JNJ and GSK are clearly negative indicators. These results could have been indicative of the need to further investigate the mechanism of these compounds before any further progression down a clinical pipeline.

### 4.3 Current use and impact of FOB/Irwin studies

Current overall CNS safety screening strategy usually includes *in vitro* techniques like brain slice electrophysiology, neuronal cell lines, multielectrode array assays and blood-brain barrier models followed by GLP first in human (FIH)-enabling FOB/Irwin studies either as a standalone study or as an integrated assessment in a repeat dose toxicology study (Jackson et al., 2019; Authier et al., 2016). In certain cases, a non-GLP FOB/Irwin study or an investigative locomotor assay is performed in addition to the previously mentioned assays and specific safety pharmacology models (e.g., EEG studies).

However, in a review to assess the utility of core battery safety pharmacology testing that included 104 FIH packages, the vast majority (78%) of FOB/Irwin studies showed no CNS effects (Baldrick, 2021). Similar findings were reported from a review of industry practices for pharmacology neurofunctional testing, where no effects were seen in approximately 62% of rodent FOB/Irwin tests (Jackson et al., 2019). Both investigations indicated that the findings in the GLP FOB/Irwin tests had no obvious impact on the safety/risk assessment of the drug. Any CNS-active drug would be anticipated to show activity in FOB/Irwin testing but this would typically be explained as expected or exaggerated pharmacology. In general, questions are likely to be raised only if a CNS-active drug failed to show effects in FOB/Irwin studies. Alternatively, it has also been reported that in a study of 50 non-CNS targeted drugs, there were widespread effects in FOB that were minor in nature and did not impact on stop-go decision making (Redfern et al., 2005). Overall, this strongly suggests, regardless of outcome and regardless of whether the drug is intended for CNS use, the FOB/Irwin studies provide minimal impact on the CNS safety assessment and further progression of (clinical) drug development. Furthermore, the minimal contribution of such studies opens the door to alternative means of CNS screening as envisaged in ICH S7A (ICHS7A, 2001) and implied in other regulatory guidance such as ICH M3 (R2) (ICH, 2009) and ICH S6 (ICH, 2011). With the above in mind, we consider the main uses and advantages of HCA over FOB/Irwin in both non-GLP and regulatory GLP scenarios.

### 4.4 Potential use and advantages of non-GLP HCA studies

The HCA may be incorporated as a routine CNS screening strategy early in development prior to GLP FIH-enabling studies as a bridge between *in vitro* assays and the *in vivo* GLP FOB/Irwin studies or as a replacement for non-GLP FOB/Irwin studies. The observations from continuous cage monitoring can be followed up with alternative behavioural assays to further investigate any positive signals. On the other hand, if the observations are clean, the potential drug candidate can be confidently progressed to the next stage of development. This scenario provides the opportunity to mitigate the risk, if any, early in development and integrate behavioural observation studies later into a GLP toxicology study to adhere to the current ICH S7A regulatory guidelines, rather than performing a standalone Irwin study (as described below). The authors understand, though, that many groups prefer standalone GLP FOB/Irwin studies as these are usually the only *in vivo* neurofunctional assessments in the life cycle of a drug candidate. Adopting HCA screening early in development as described could provide confidence to these groups that Irwin assessments can be successfully incorporated into other GLP studies. This could be especially valuable when investigating a novel modality, for which a body of evidence establishing the potential CNS risks does not already exist.

Performing alternative CNS de-risking studies as a follow up to the HCA assessments will enable the drug development team to make go/no-go decisions early, which again will help to avoid unnecessary *in vivo* studies. For instance, if an unmitigable seizure-like phenotype is identified early (for example, from the HCA video), the candidate molecule can be retired or replaced without performing additional animal studies. In the current testing scheme, these risks are usually identified following the GLP FOB/Irwin requiring follow up definitive studies which would prolong the drug development process and increase the number of animals required overall per compound.

Application of HCA in a non-GLP setting (and sharing these data publicly) may also make future acceptance of the model within a regulatory framework smoother. The present study should go a considerable way to demonstrating the advantages of HCA (longitudinal recording, including in the dark phase, of normal, group-housed animal behaviour with minimal technician/scientist intervention) over conventional behavioural observation and we hope that this will encourage establishment of the method within industry and contract research organisations (CROs) as an early screening tool. There would be a cost implication if HCA was used for additional investigational studies rather than as a whole or partial replacement for FOB/Irwin. However, we would argue that the superiority of HCA in enabling early go/no-go decisions will reduce costs overall by avoiding the progress of unsuitable candidates further down the development pipeline and into costly animal studies, e.g., GLP FOB/Irwin studies.

### 4.5 Potential use and advantages of GLP HCA studies

The second scenario is the incorporation of HCA into GLP repeat dose toxicity studies instead of performing CNS assessment in a standalone GLP study. Using a weight of evidence approach, where non-GLP in-life observational data (e.g., FOB/Irwin, HCA, clinical signs from investigational toxicology studies), combined with other *in silico* and *in vitro* assays (as previously mentioned) and the HCA in a GLP repeat dose setting, gives a more robust overall CNS assessment. A tiered approach as suggested by Redfern et al. (2019), could be considered, where candidate drugs are selected according to CNS penetration. If a drug does not enter the CNS, the overall testing strategy can be tailored accordingly, thus reducing the total number of animals used for CNS safety assessment.

The integration of FOB/Irwin into repeat dose toxicology studies affords advantages, which have been well documented in the literature (Redfern et al., 2013), however this is practically difficult to achieve in an appropriate environment with minimal disturbance to animals, due to other activities that are necessary to fulfil the primary objectives of the study (e.g., blood collections, clinical observations). We show here that the HCA can be used successfully in a toxicology study environment, where animals are dosed daily for 28 days and data collected for 4 days prior to and throughout the duration of the study. While it would technically be possible to perform FOB/Irwin measurements over an equivalent timeline, this would entail a greater burden in terms of both human resource, and animal interventions. Aside from the practical advantages of incorporating HCA in GLP toxicity studies, continuous neurobehavioral data can be collected which is superior to that collected via standard FOB/Irwin at limited time points, and the reduced burden on individual animals represents an animal welfare refinement over current practice.

Adoption of this second scenario is more challenging, relying not only on its availability, but also its validation since validation of computerised systems for use on GLP studies is required by the OECD Principles of GLP (OECD, 1998) and FDA 21 CFR Part 11 (FDA, 2003). Regulatory acceptance is not in itself a barrier as discussed above in relation to ICH S7A requirements, but a test case and increased awareness of HCA’s abilities would build confidence among all stakeholders, including the regulatory sector. A recent industry survey (Jackson et al., 2019) showed that the majority of GLP safety assessment studies are outsourced to CROs. Investment in HCA equipment by CROs will be driven by demand, but conversely, if the equipment is not available and HCA is not offered as an option for GLP CNS assessment, the tendency will be to preserve the *status quo* and perform conventional CNS assessment. As outlined above, inclusion of HCA versus FOB/Irwin in a GLP toxicity study has many practical advantages. These practical aspects, alongside the scientific evidence of HCA utility presented here, may encourage investment and uptake of HCA by CROs as a viable alternative service offering to standard methods.

In either of the above scenarios, the suitability or superiority of HCA as a replacement rests on the greater sensitivity noted in the present studies and in its ability to assess responses to routine handling interventions (e.g., blood sampling, (see Figures 5D,E), and cage changes (Tse et al., 2018). While it does not provide identical behavioural measures to FOB/Irwin studies (Redfern et al., 2019), the latter are rarely if ever used to inform decision making, so such an argument seems rather pointless. Similarly, the question of whether HCA observations are as, or more, clinically relevant than those from FOB/Irwin testing is meaningless if the latter does not sufficiently inform clinical development.

## 5 Conclusion

Given that all three compounds had been reported to show no serious effects in previous FOB/Irwin studies, the extent and strength of behavioural changes observed using HCA was remarkable. In addition to non-evoked behavioural profiles, we observed evoked responses in the home cage to events such as environmental changes and animal handling. While the preceding figures captured illustrative examples of individual behavioural parameters within specific time windows, these inevitably represent a subset of the detailed longitudinal profiles captured from these animals (see Supplementary data). It is therefore notable that many of the effects observed were temporally consistent over multiple hours, and often spanned multiple parameters (e.g., rearing, locomotion and temperature all being affected in some cases). The absence of such effects in some of the earlier modified Irwin studies reflect a functional divergence between the sparse observational snapshots used in the modified Irwin, and the detailed temporal integration afforded by continuous monitoring. It may also reflect a further, more fundamental, difference in the underlying experimental premise–subjective observations of behaviour recorded in the presence of operators, compared to non-evoked measurement in the home cage.

## 6 Implications for the 3Rs

Application of the HCA has important benefits for animal welfare and the 3Rs (Replacement, Reduction and Refinement of animals used in research). The present study demonstrates the potential of HCA to improve compound selection and prevent unsuitable molecules progressing into more intensive animal work and clinical development. The extent of reduction in animal numbers is impossible to quantify precisely but the fact that the HCA results gave rise to concerns with two of the three compounds examined suggests that use of the HCA could have a considerable effect on the numbers of phase I failures with a corresponding reduction in non-productive animal use.

In a standalone scenario, while the HCA uses around the same number of animals as FOB/Irwin testing, the larger amount of data per animal is in itself a reduction and gives rise to more robust and less variable findings. The greater compatibility of HCA with repeat dose toxicology studies should encourage the integration of HCA in favour of FOB/Irwin and discourage use of standalone GLP FOB/Irwin testing with a saving in animal numbers.

Group housing and continuous non-invasive monitoring are substantial refinements that reduce the potential for stress-induced behaviour changes which may mask the impact on animal behaviour of the compound being tested (Gouveia and Hurst, 2017). Compared to integration of FOB/Irwin in a repeat dose toxicology study, HCA is practically much easier to combine with the other activities in the study with less impact on animal behaviour, reduced study complexity and reduced technician time for in-life procedures. Furthermore, the greater sensitivity of the HCA compared with FOB/Irwin could lead to more precise dose setting for repeat dose studies and avoid the use of unnecessarily high and scientifically irrelevant doses that might have adverse effects on animal welfare.

## Data Availability

The original contributions presented in the study are included in the article/Supplementary Material, further inquiries can be directed to the corresponding authors.

## Footnotes

1 It is worth noting that the study itself required implementation of standard animal welfare protocols (such as bedding changes), and pharmacokinetic measurements in the form of blood draws. Home cage monitoring nonetheless presents an opportunity to measure any evoked response resulting from these interventions.

## Author’s note

ICON Plc was formerly known as Charles River Laboratories.
