# Supplementary material for: Rodent home cage monitoring for preclinical safety pharmacology assessment: results of a multi-company validation evaluating nonclinical and clinical data from three compounds
Source: Front Toxicol. 2025 Nov 5;7:1655330. doi: 10.3389/ftox.2025.1655330 (PMC12626872; doi:10.3389/ftox.2025.1655330)
Supplement: Supplementary file 1 [file DataSheet1.zip › Supplementary_Data_Overview.pdf]

Supplementary Data

This document accompanies supplementary data for all recordings conducted during Phase 2 (compound phase) of the present study, in particular: a ZIP file containing three subfolders (one per compound), each containing a set of CSV files with detailed measurements from all corresponding recordings (for each compound there are 40 CSV files: 2 cage groups x 4 dose levels x 5 parameters). This document also enumerates any irregularities that need to be considered when analysing this data.

CSV File names: Files are named according to the following convention:

COMPOUND_MEASUREMENT_DOSE_CAGE-#.CSV

**COMPOUND**	Which of the three compounds (AZ, GSK, JNJ) the data corresponds to.
**MEASUREMENT**	Measurement type contained in the file (see below for information on units).
**DOSE**	The dose level in mg/kg or “VEH”, which denotes the Vehicle control.
**CAGE-#**	The cage group. It should be noted that – within a given compound – all recordings with the same cage group designation (e.g. Cage-1) were recorded concurrently.

CSV File structure: Each CSV file contains 5 columns, described as follows (with each row thereafter representing a 10 minute time bin):

**TIMESTAMP**	Start time + date of current bin in format “YYYY-MM-DD HH:MM”
**DAY INDEX**	Number representing each 24hr period (delineated by the start of the 12 hr lights-on phase at 7am), relative to dosing day. Dosing day (where the dosing event occurs at 10am) has an index of 0, while days recorded before/after dosing day have a negative/positive index respectively.
**RFID# (x 3)**	Each of the remaining 3 column headings correspond to the unique RFID tag number of each animal within the cage group. The values in each column correspond to the parameter specified in the file name (e.g. Distance) recorded in 10 minute bins (sufficient to regenerate all plots and analysis presented in the paper).

Units: All measurements correspond to a mean value over the duration of a 10 minute bin (CSV files) or a 60 minute bin (plots in PDF files). Any units corresponding to activity (i.e. distance, rearing, drinking) are normalised on a “per minute” basis to facilitate comparison over different temporal aggregations.

**DISTANCE**	cm / minute	(Distance travelled.)
**TEMPERATURE**	Celsius	(Subcutaneous body temperature.)
**SEPARATION**	cm	(Average distance to closest cage-mate.)
**REARING**	seconds / minute	(Time spent rearing.)
**DRINKING**	seconds / minute	(Time spent drinking.)

Irregularities / Exclusions: Any known cases of data that was missing, irregular – or gave cause to be excluded from subsequent analysis – are enumerated below, for each of the three compounds in the study.

AZ All Cage-1 recordings were terminated five days early, on the first day after dosing (Day Index: 1) due to operator misunderstanding.

The recording [8.5mg/kg, Cage-2] was considered unreliable from the second day after dosing (Day Index: 2) onwards, due to power supply failure of the RFID reader. This was detected due to a sudden decrease in the typical RFID read frequency in the raw data.

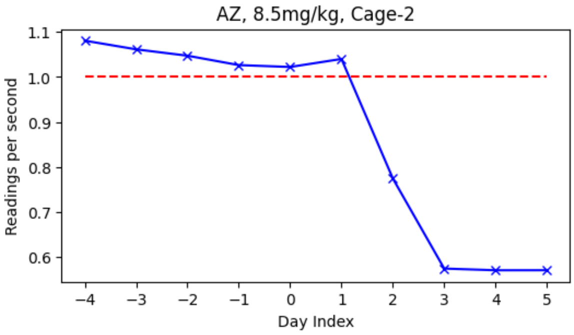

GSK Abnormally high rearing levels were observed for the recording [Vehicle, Cage-1] during the dark phase of the fourth and fifth days after dosing (Day Index: 4 & 5). Manual inspection of the video footage revealed that the cage lid had not been fully closed, and that the increased rearing behaviour corresponded to the rats spending time looking through the opening between cage body and lid.

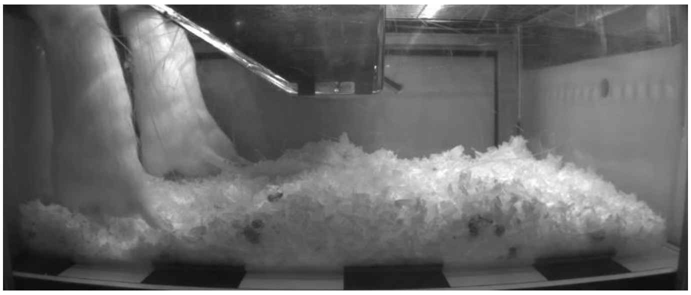

JNJ Temperature data for the following animals IDs was considered unreliable due to abnormally high variance in readings (the sign of a failed temperature transponder in an RFID chip):

985141003627903	[10mg/kg, Cage-1]
985141003627912	[10mg/kg, Cage-1]
985141003627856	[Vehicle, Cage-2]
985141003627859	[10mg/kg, Cage-2]

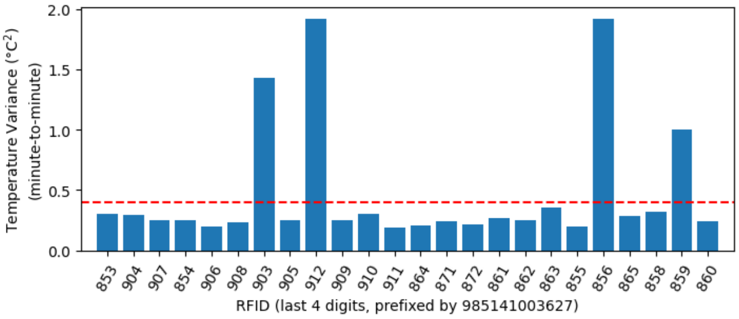

Temperature data for these chips was therefore excluded from all temperature analysis presented in the paper (with N numbers adjusted accordingly in any downstream statistical tests).
