# Supplementary material for: Rodent home cage monitoring for preclinical safety pharmacology assessment: results of a multi-company validation evaluating nonclinical and clinical data from three compounds
Source: Front Toxicol. 2025 Nov 5;7:1655330. doi: 10.3389/ftox.2025.1655330 (PMC12626872; doi:10.3389/ftox.2025.1655330)

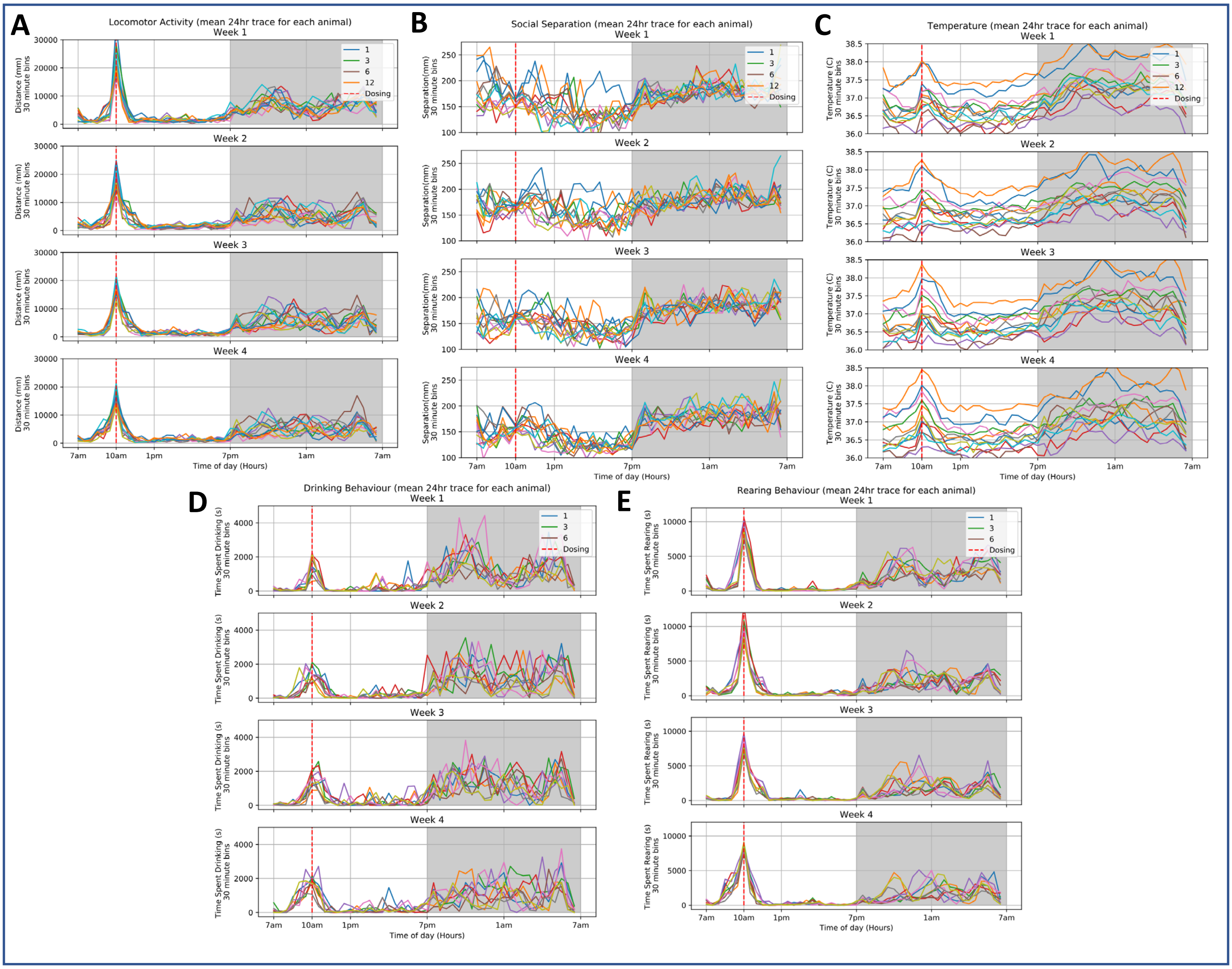

Supplementary Figure 1.

We validated that the technology could run for up to four weeks and hence suitable for a toxicology deployment. Rats were orally dosed with saline at 10am every day. For each of the four weeks we averaged the seven days to produce a daily average trace per animal for each of the measured parameters including (left to right), locomotor activity, social interactions, temperature, drinking and rearing (see also Figure 1b).

Compound	Irwin		HCA
**JNJ (mg/kg)**	Mean plasma levels (ng/ml), 1h and 3h post-dose		Mean plasma levels (ng/ml), 2h post-dose
	1h	3h	
**10**	533	329	631
**40**	3453	3657	3367
**300**	9437	6780	6010
**GSK (mg/kg)**	Mean plasma levels (ng/ml), 4h post-dose		
**10**	69*		225
**50**	911*		1281
**150**	Not tested		2640
**AZ (mg/kg)**	Mean plasma levels (ng/ml), 24h post-dose		
**8.5**	642		729
**28**	2203		2325
**85**	5400		5514
*** Values were generated in a separate GLP toxicology study**			

Supplementary Figure 2. Mean plasma levels (ng/ml) of JNJ, GSK and AZ measured in Irwin and HCA studies

Supplementary Data: AZ – Distance (cm / min)

Average 24-hour traces (mean ± S.E.M) for each day of the study:

- Sample size is n=6 from start until end of “Dosing Day + 1”; n=3 thereafter.
- Data for 8.5 mg/kg dose unreliable from “Dosing Day + 2” onwards.

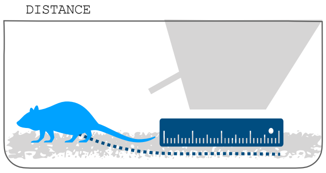

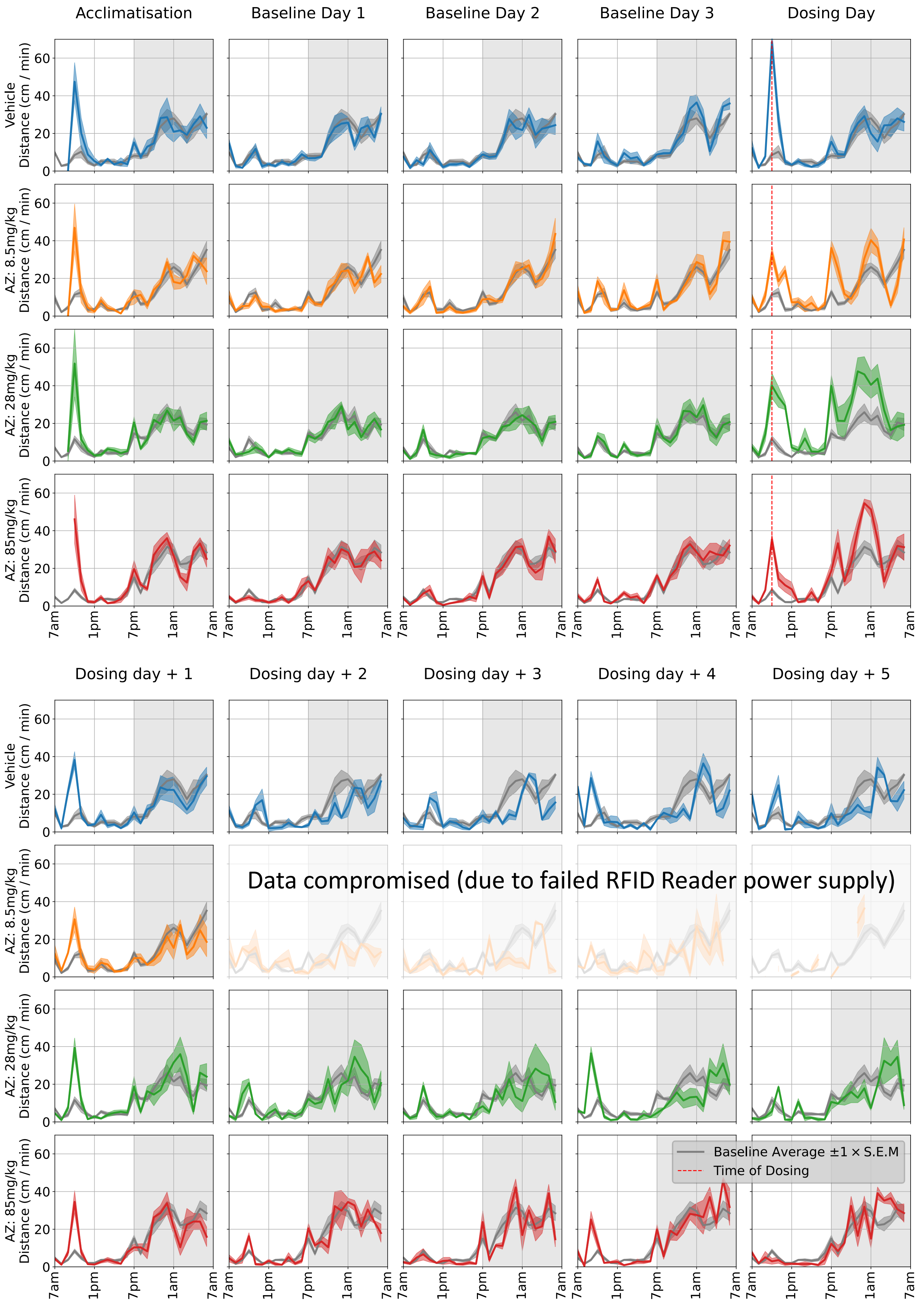

Supplementary Data: AZ – Rearing (s / min)

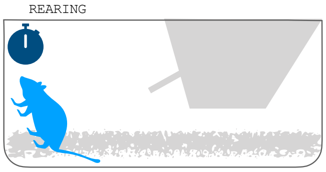

Average 24-hour traces (mean ± S.E.M) for each day of the study:

- Sample size is n=6 from start until end of "Dosing Day + 1"; n=3 thereafter.
- Data for 8.5 mg/kg dose unreliable from "Dosing Day + 2" onwards.

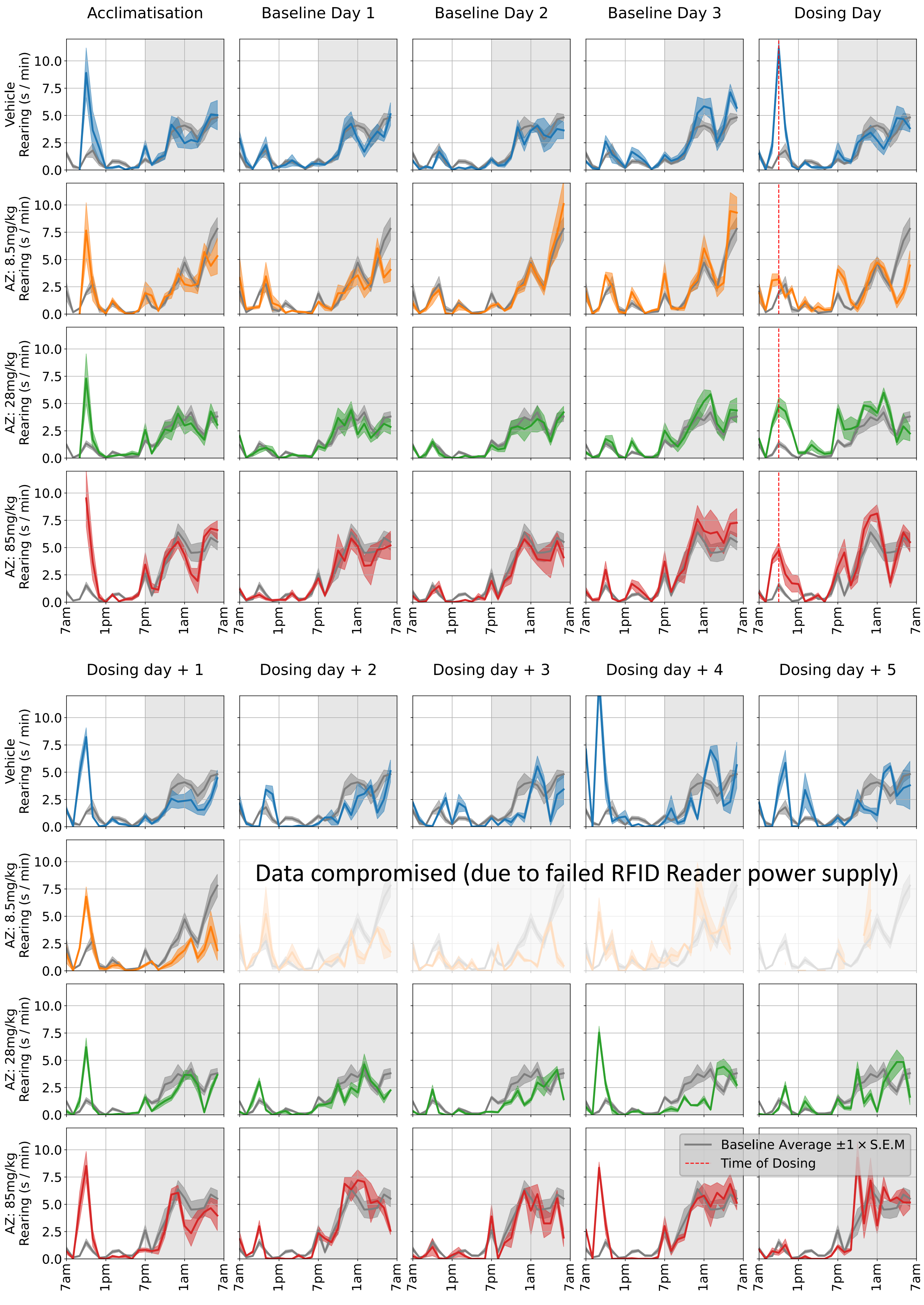

Supplementary Data: AZ – Temperature (°C)

Average 24-hour traces (mean ± S.E.M) for each day of the study:

- Sample size is n=6 from start until end of "Dosing Day + 1"; n=3 thereafter.
- Data for 8.5 mg/kg dose unreliable from "Dosing Day + 2" onwards.

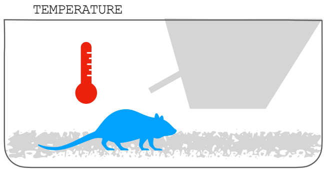

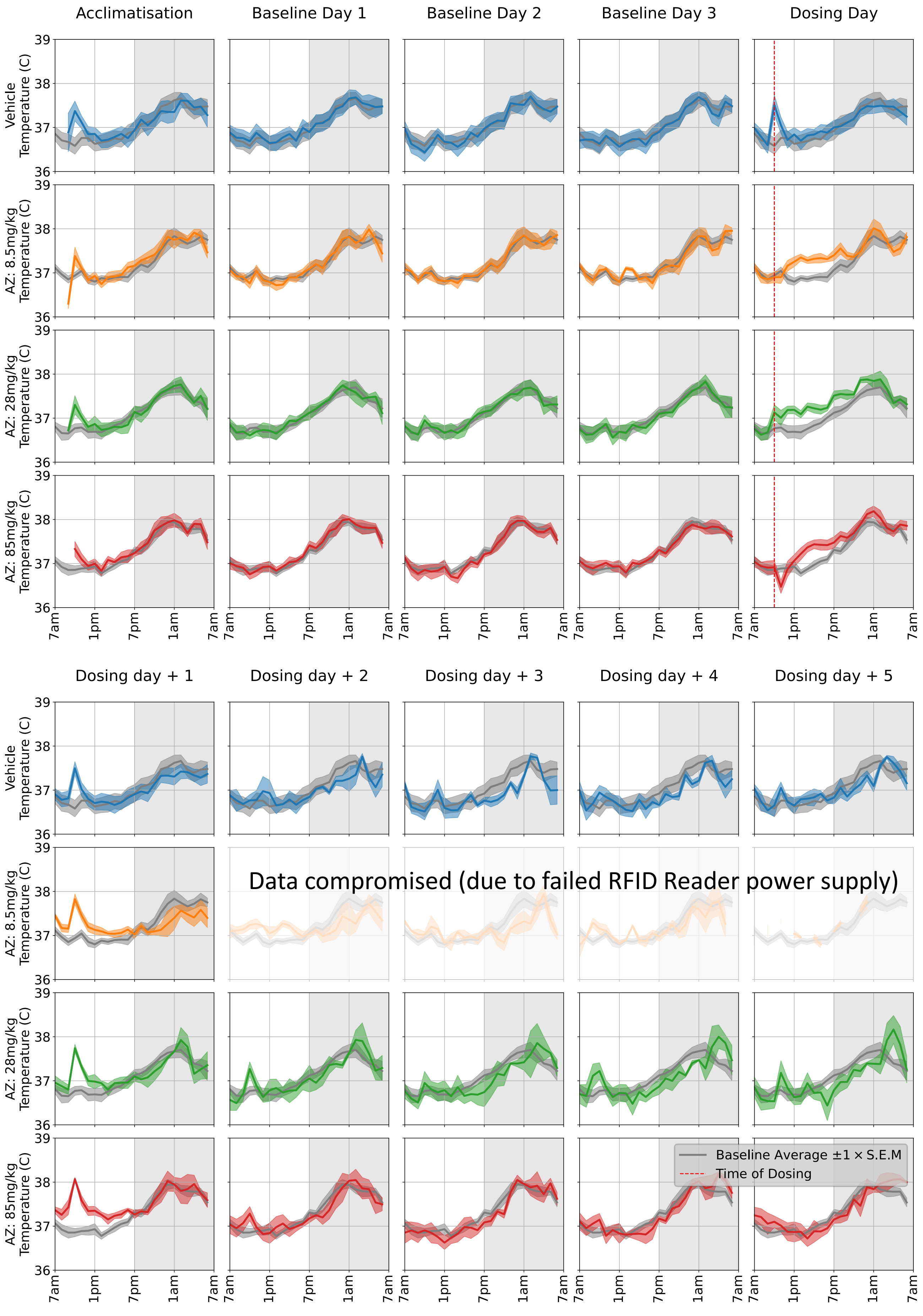

Supplementary Data: AZ – Separation (cm)

Average 24-hour traces (mean ± S.E.M) for each day of the study:

- Sample size is n=6 from start until end of “Dosing Day + 1”; n=3 thereafter.
- Data for 8.5 mg/kg dose unreliable from “Dosing Day + 2” onwards.

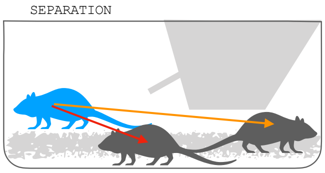

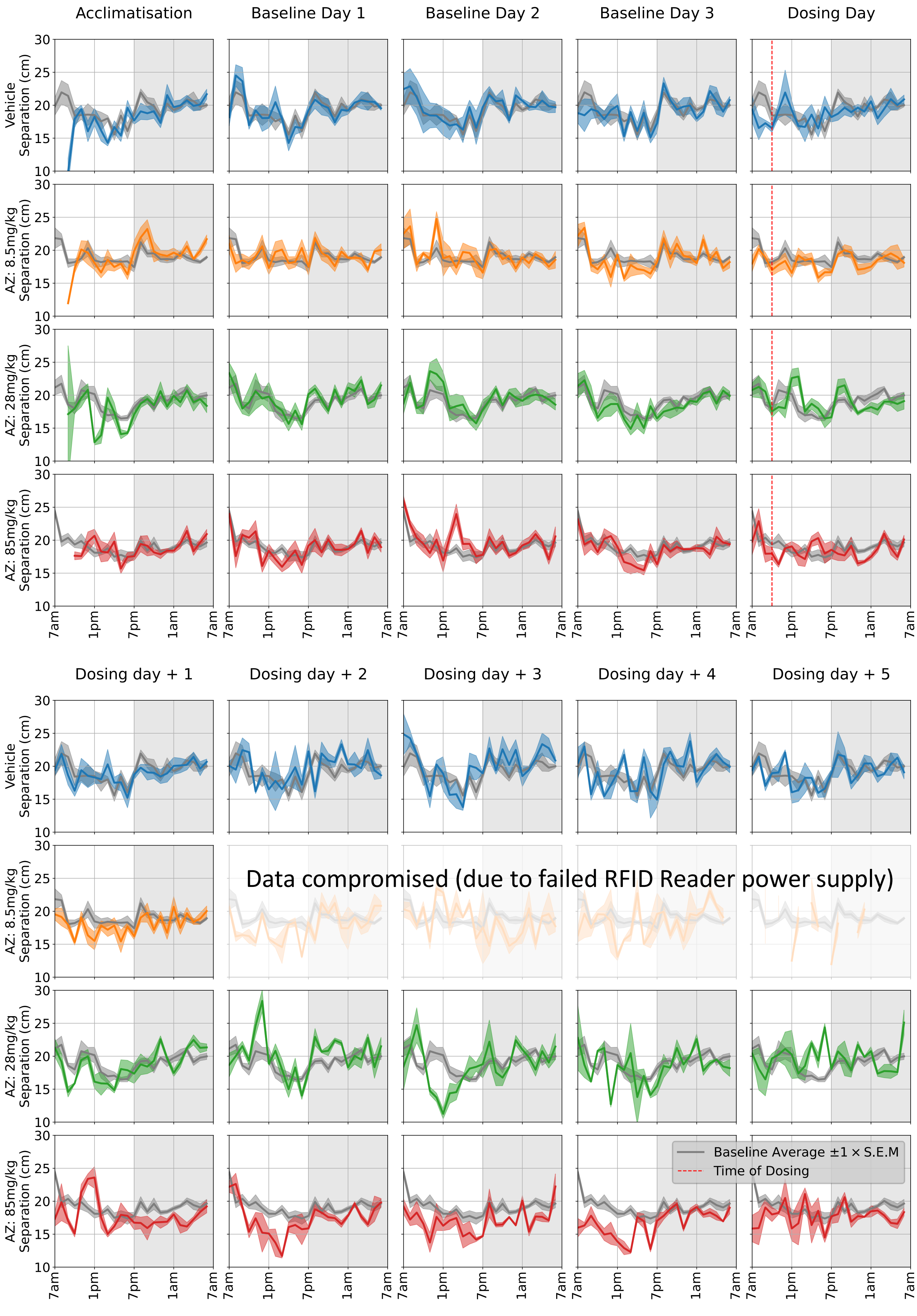

Supplementary Data: AZ – Drinking (s / min)

Average 24-hour traces (mean ± S.E.M) for each day of the study:

- Sample size is n=6 from start until end of "Dosing Day + 1"; n=3 thereafter.
- Data for 8.5 mg/kg dose unreliable from "Dosing Day + 2" onwards.

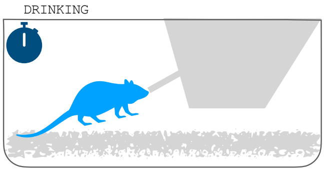

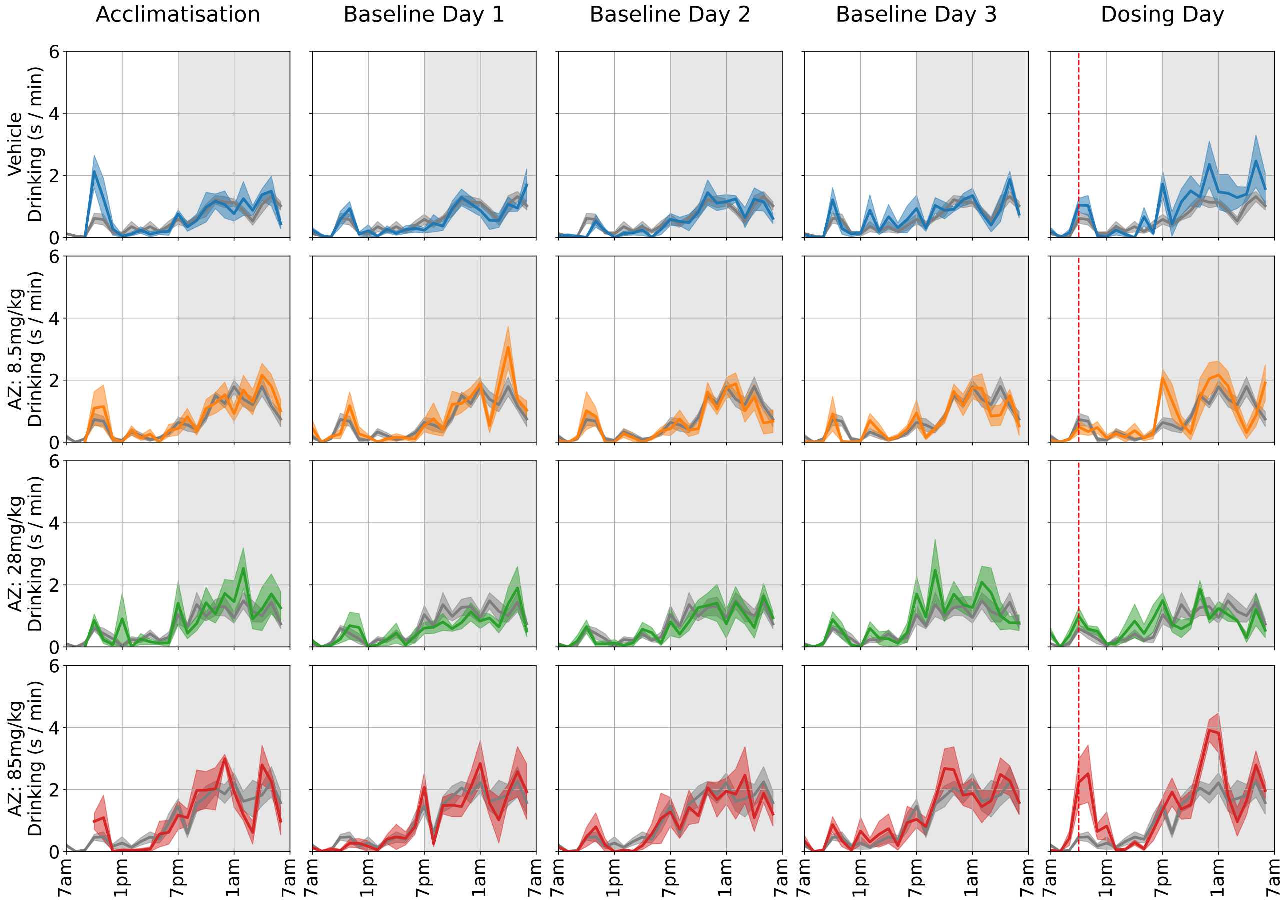

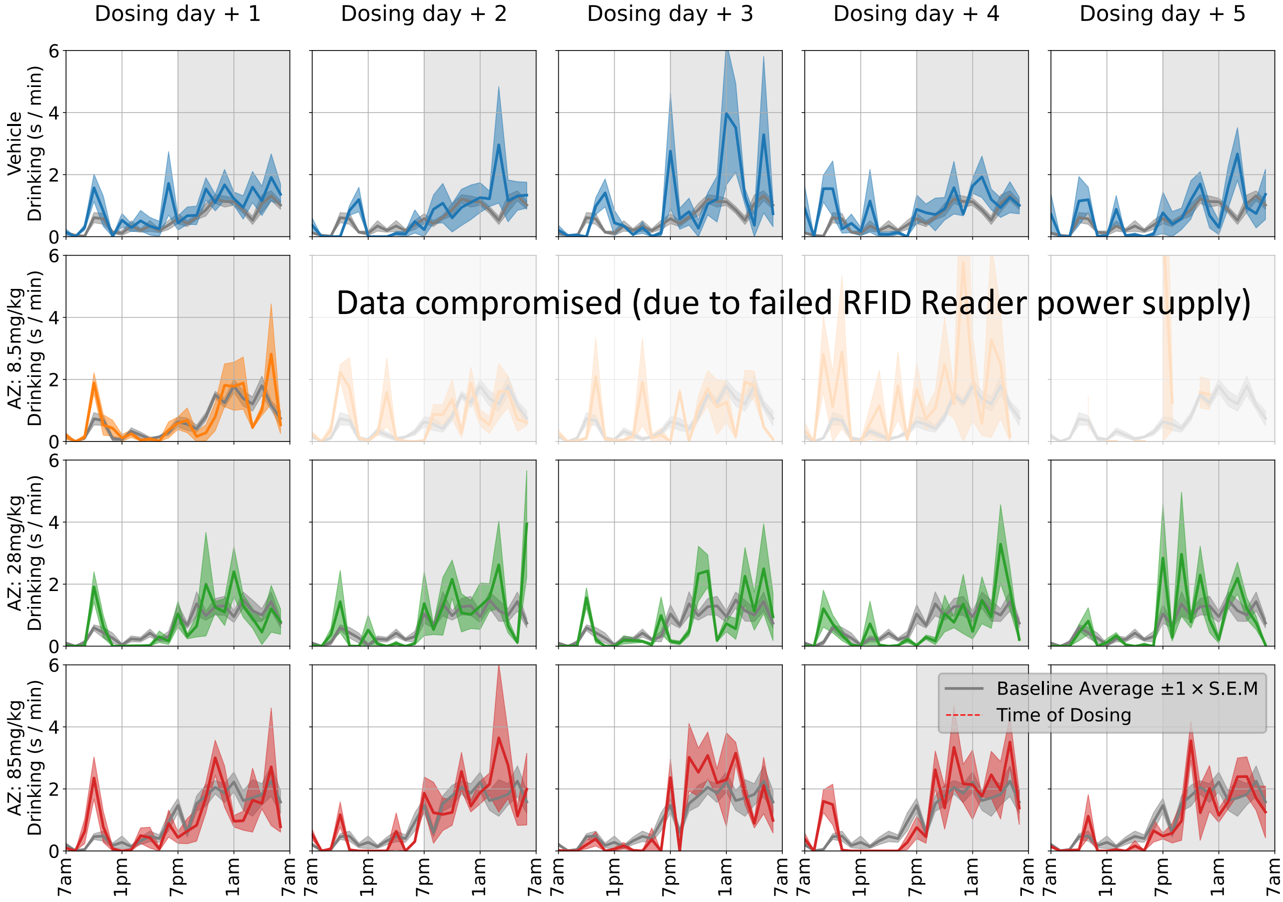

Supplementary Data: GSK – Distance (cm / min)

Average 24-hour traces (mean ± S.E.M) for each day of the study:

- Sample size is n=6 for duration of recording.

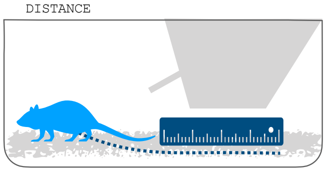

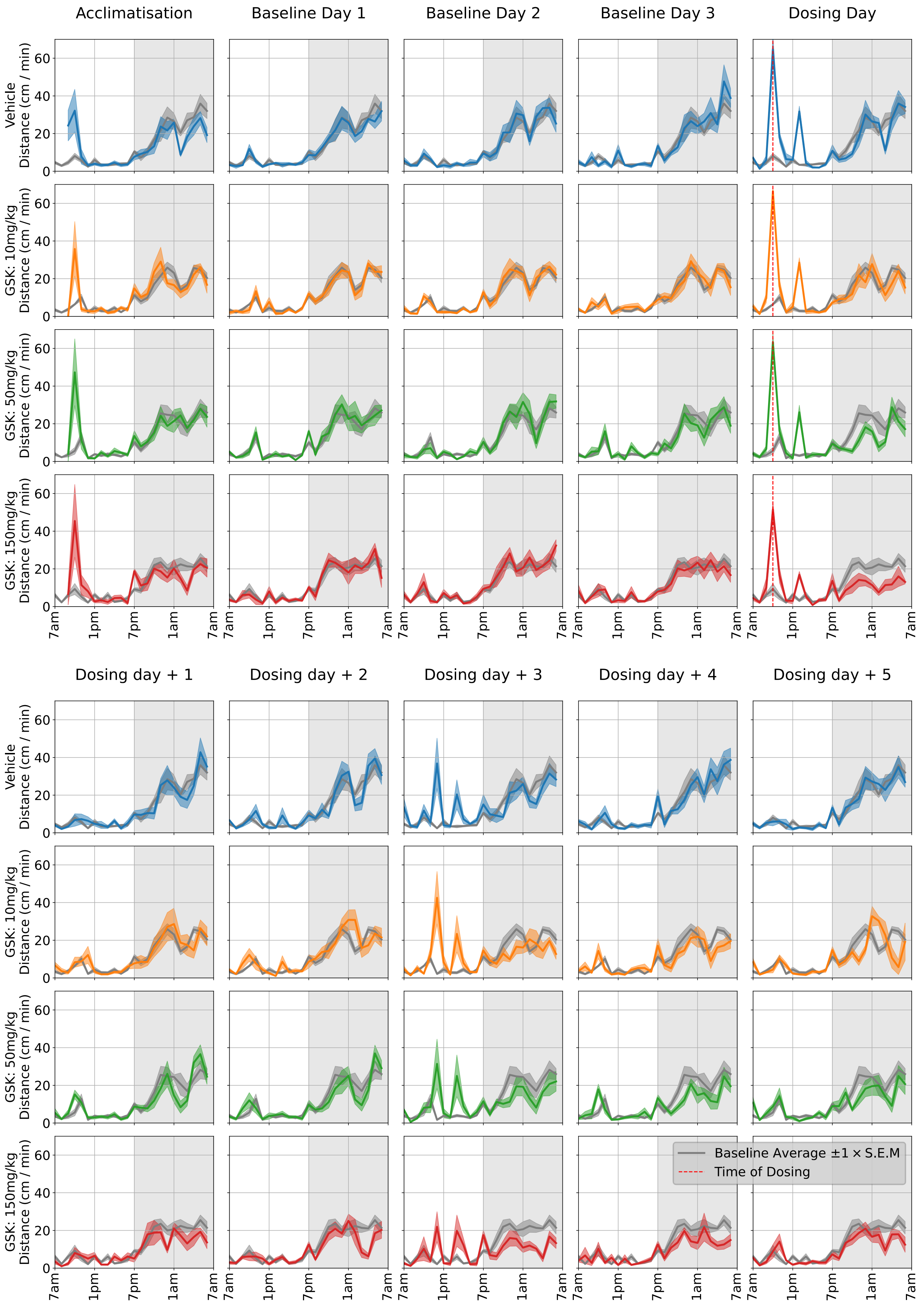

Supplementary Data: GSK – Rearing (s / min)

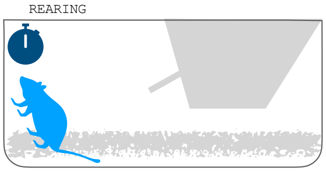

Average 24-hour traces (mean ± S.E.M) for each day of the study:

- Sample size is n=6 for duration of recording.
- Elevated Rearing in Vehicle group on “Dosing Day + 4” and “Dosing Day + 5” was caused by one cage-group of animals exploring partly-opened cage lid.

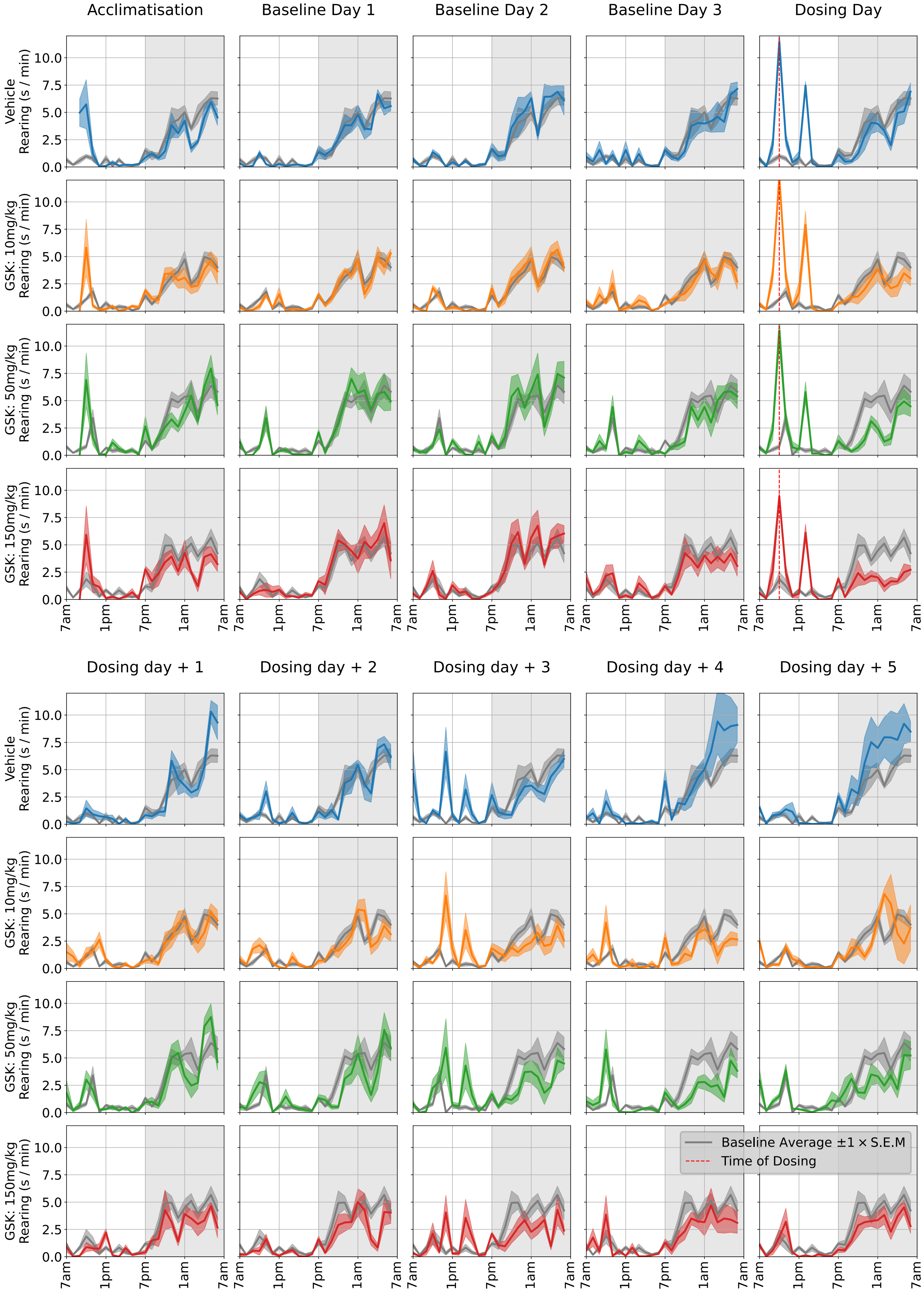

Supplementary Data: GSK – Temperature (°C)

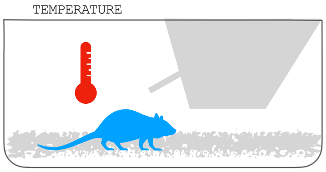

Average 24-hour traces (mean ± S.E.M) for each day of the study:

- Sample size is n=6 for all other dose levels for duration of recording.

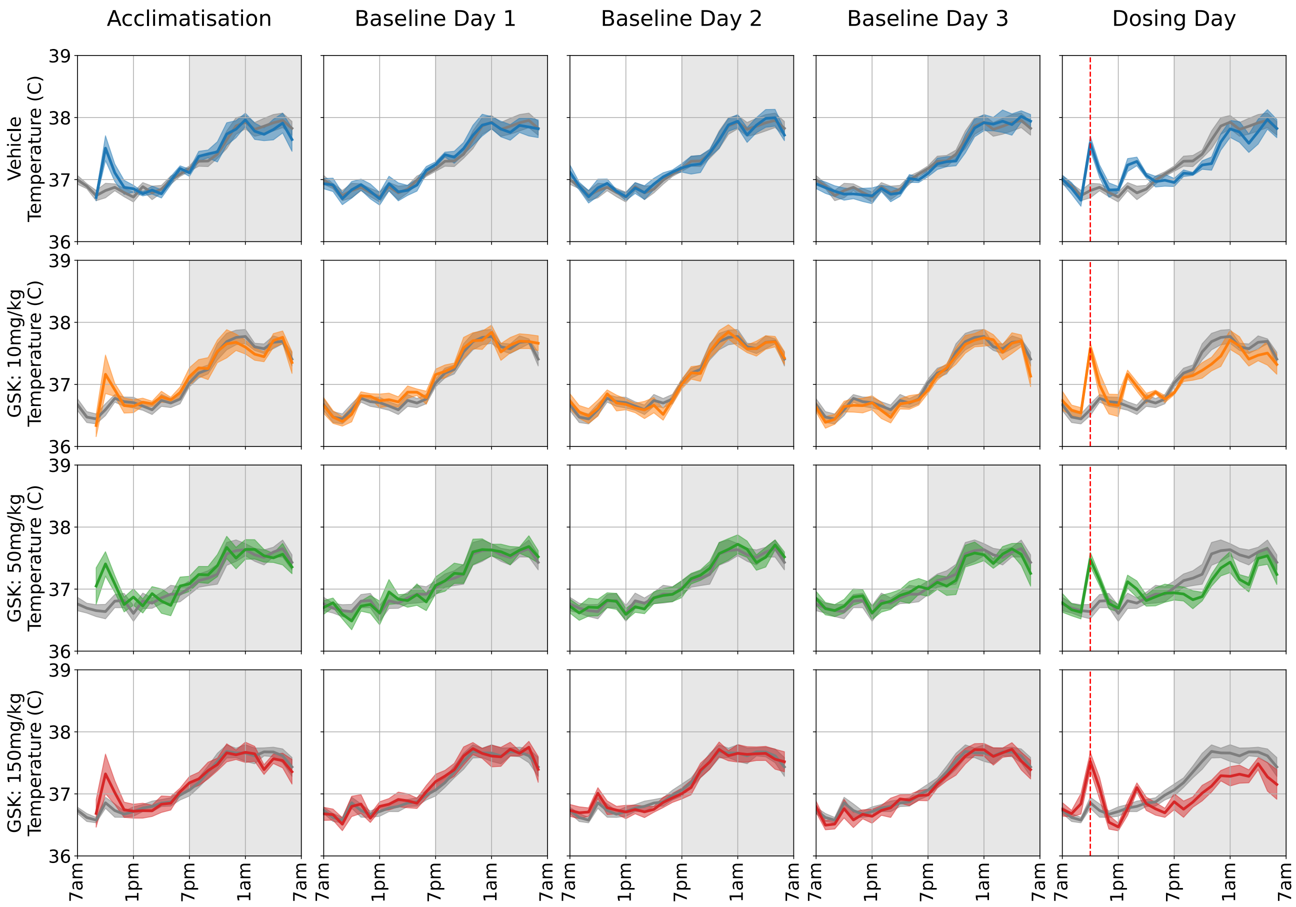

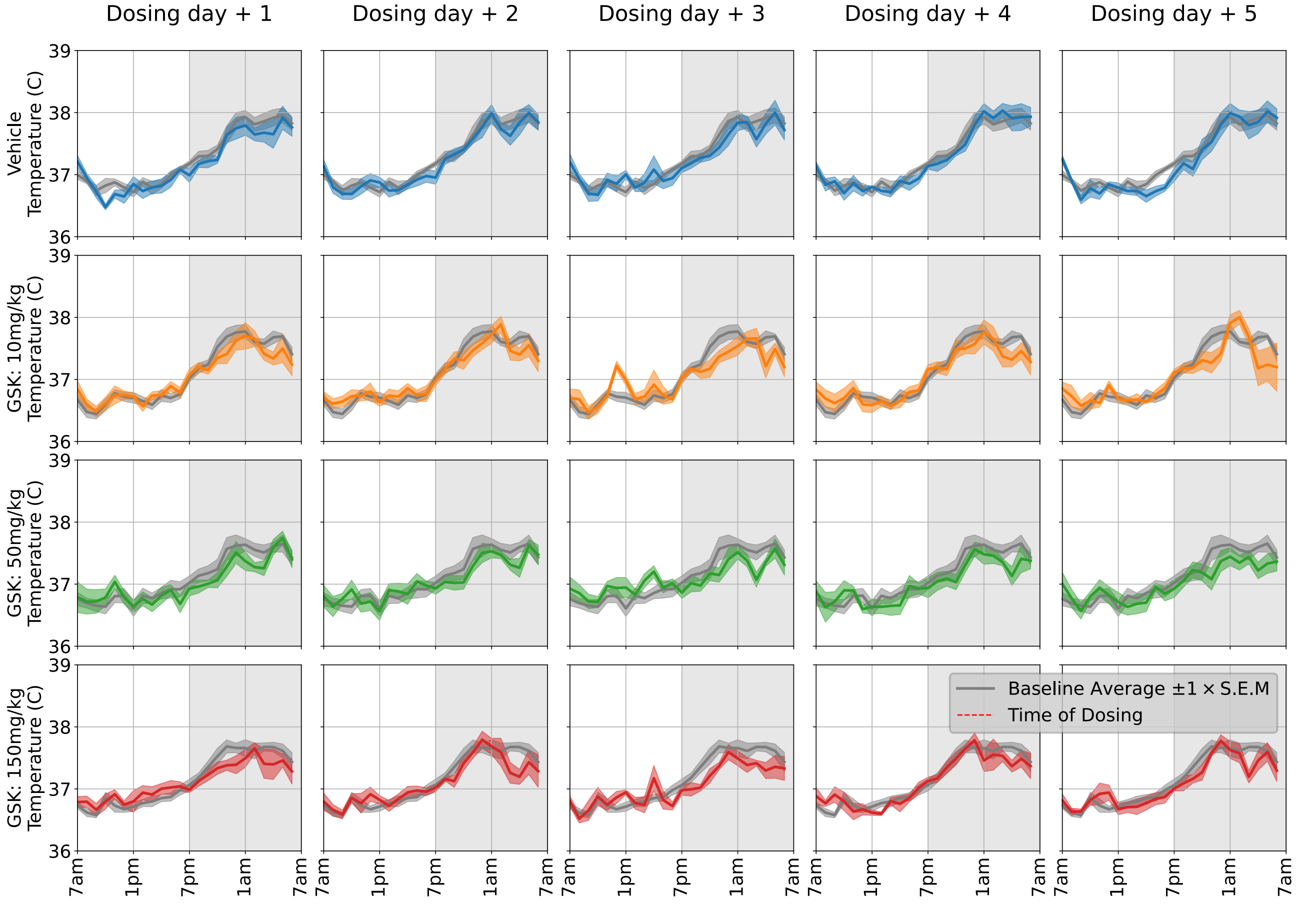

Supplementary Data: GSK – Separation (cm)

Average 24-hour traces (mean ± S.E.M) for each day of the study:

- Sample size is n=6 for duration of recording.

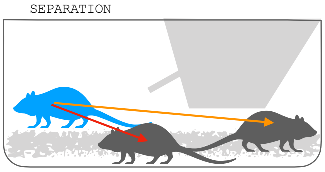

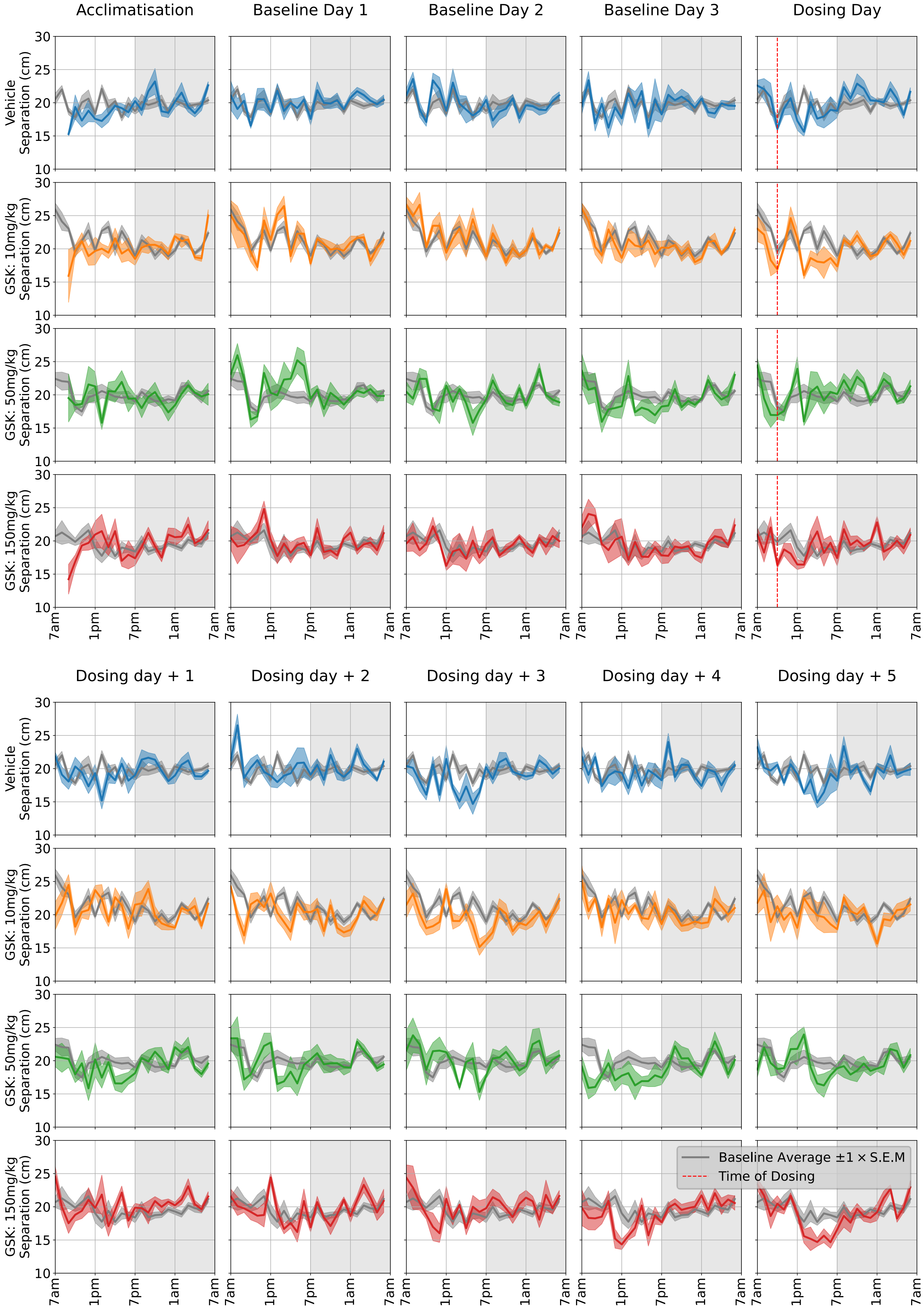

Supplementary Data: GSK – Drinking (s / min)

Average 24-hour traces (mean ± S.E.M) for each day of the study:

- Sample size is n=6 for duration of recording.

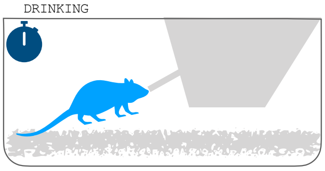

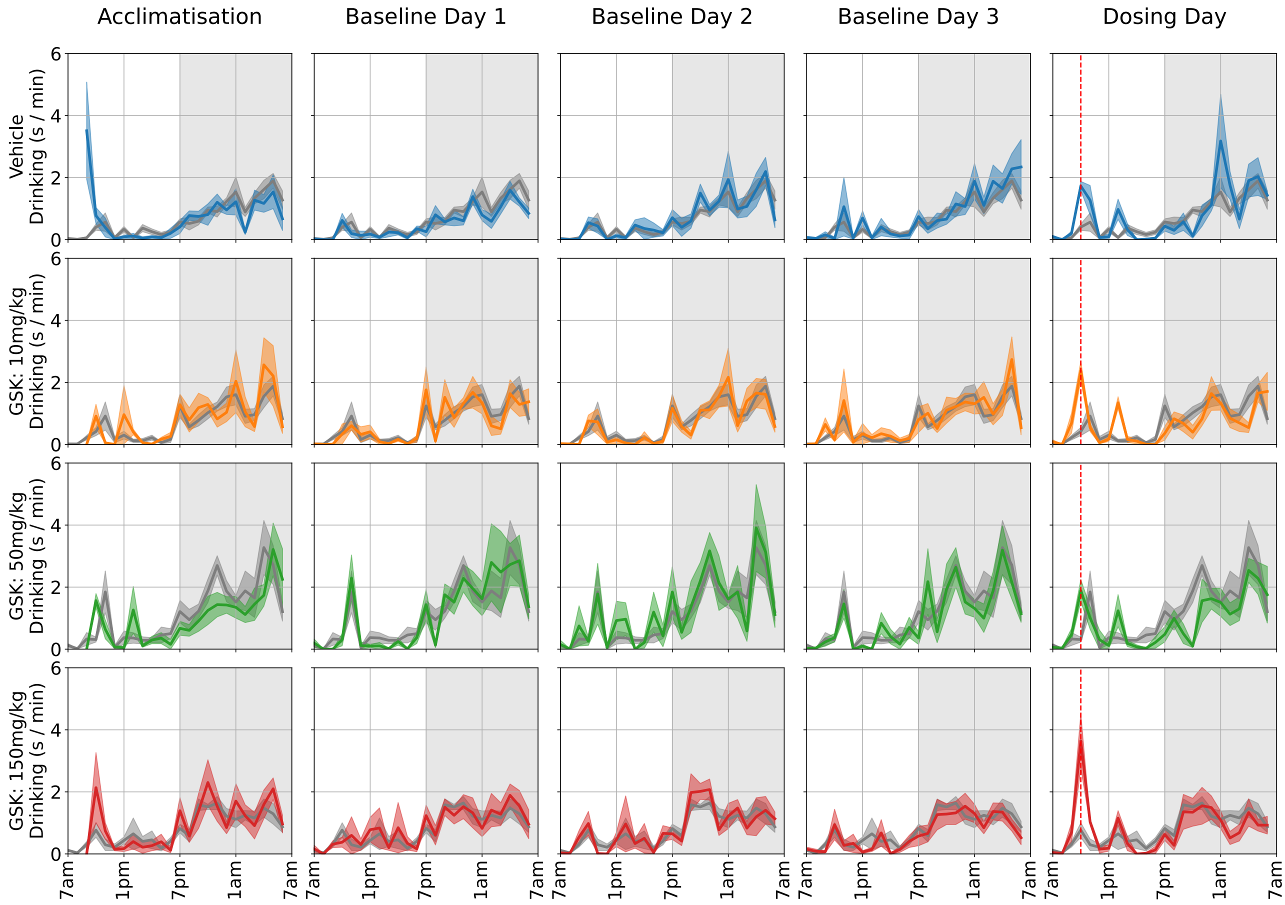

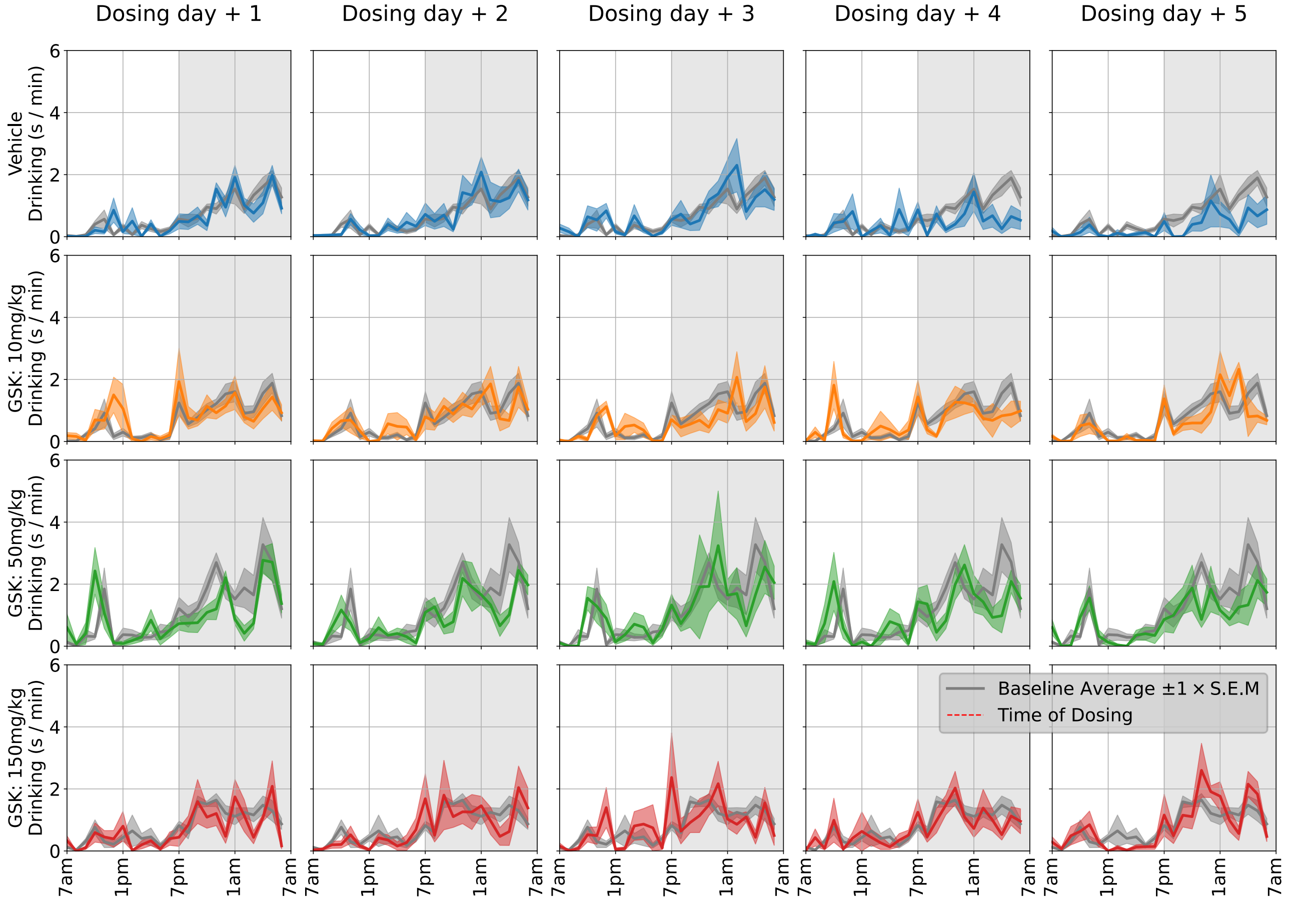

Supplementary Data: JNJ – Distance (cm / min)

Average 24-hour traces (mean ± S.E.M) for each day of the study:

- Sample size is n=6 for duration of recording.

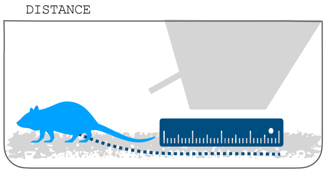

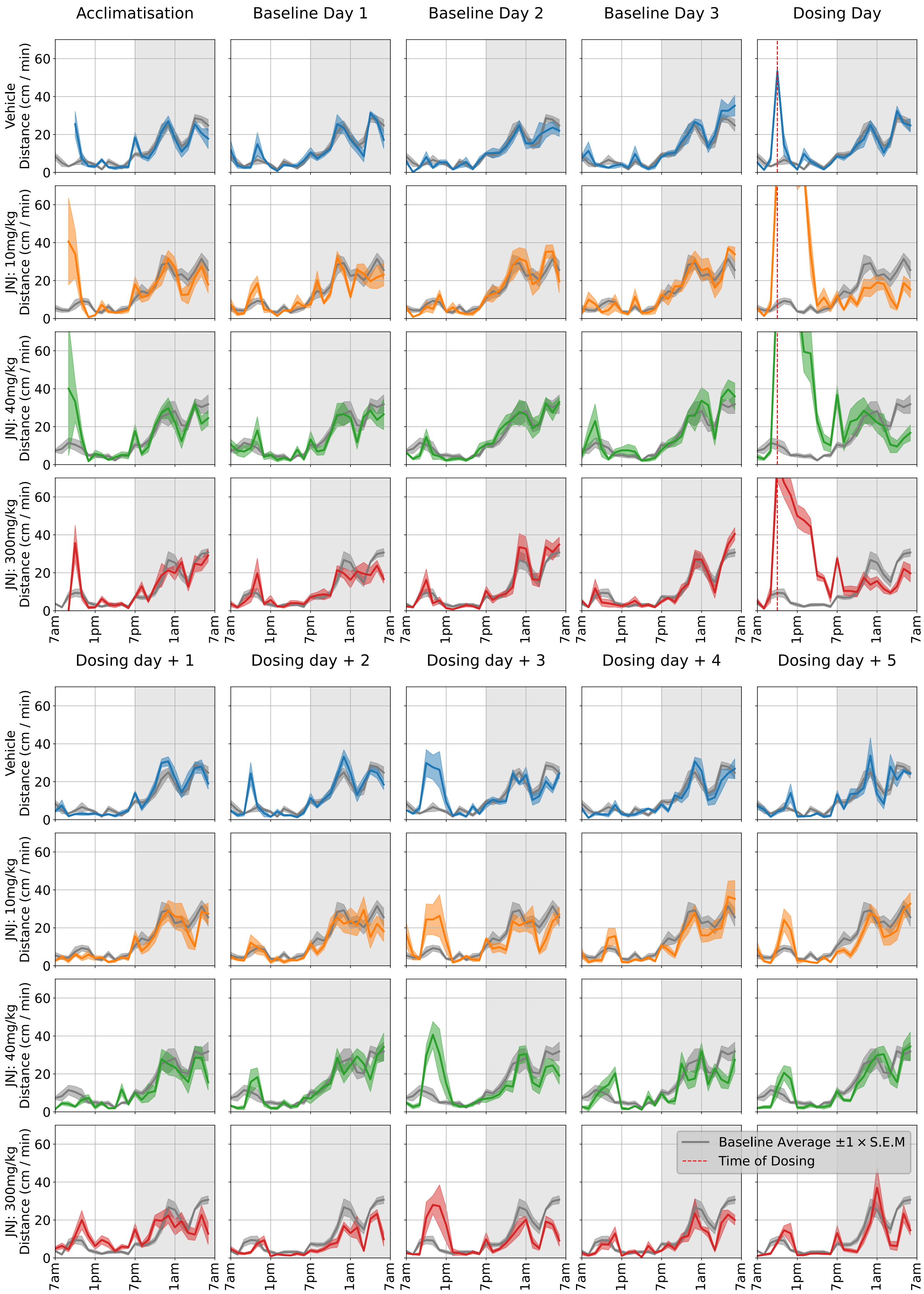

Supplementary Data: JNJ – Rearing (s / min)

Average 24-hour traces (mean ± S.E.M) for each day of the study:

- Sample size is n=6 for duration of recording.

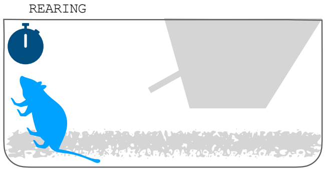

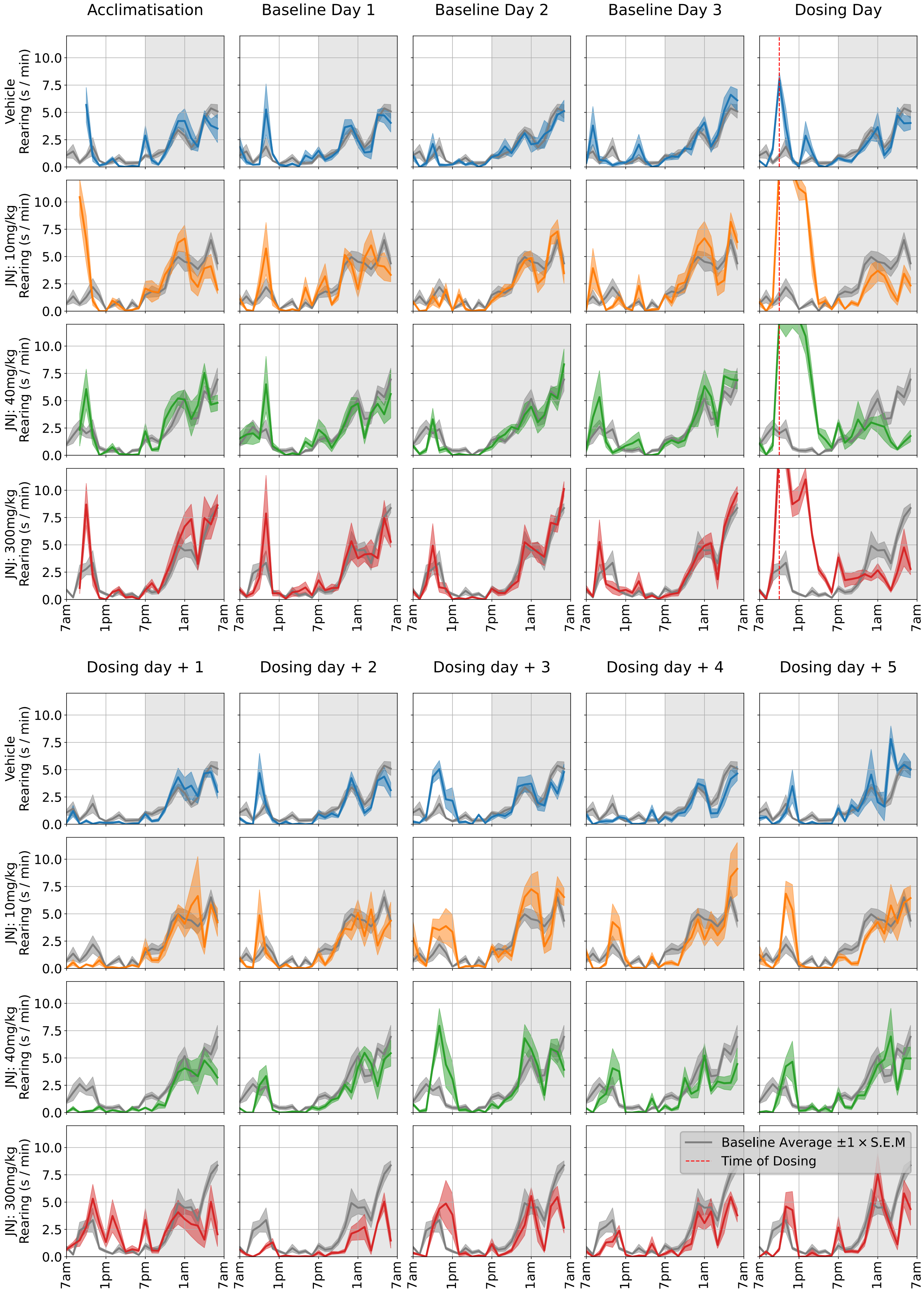

Supplementary Data: JNJ – Temperature (°C)

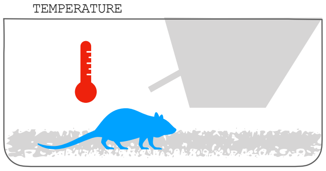

Average 24-hour traces (mean ± S.E.M) for each day of the study:

- Sample size is n=3 for 10mg/kg dose (due to 3 faulty temperature transponders).
- Sample size is n=5 for Vehicle dose (due to 1 faulty temperature transponder).
- Sample size is n=6 for all other dose levels for duration of recording.

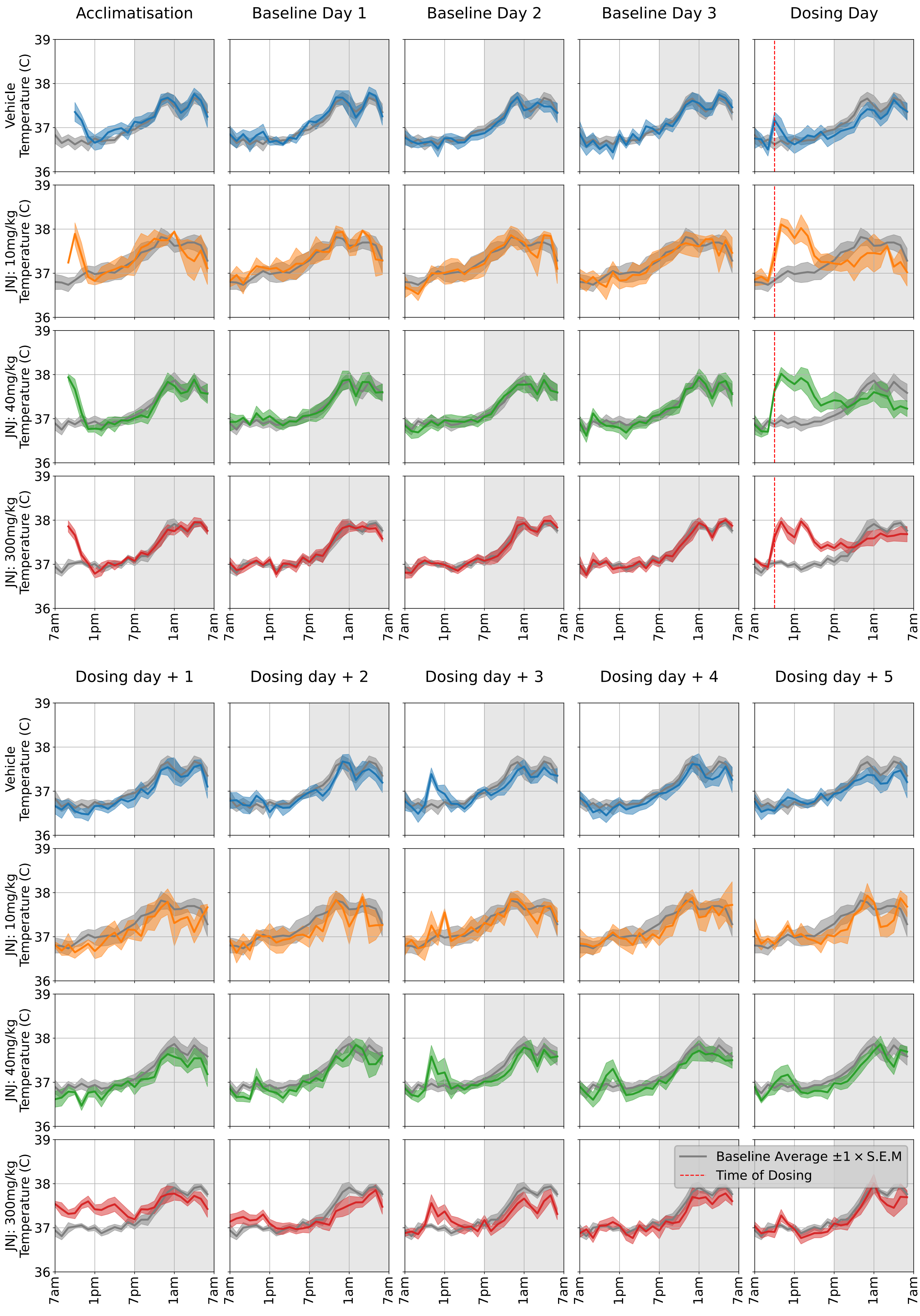

Supplementary Data: JNJ – Separation (cm)

Average 24-hour traces (mean ± S.E.M) for each day of the study:

- Sample size is n=6 for duration of recording.

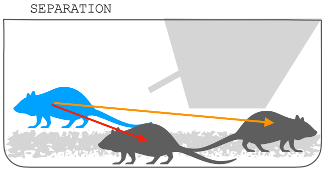

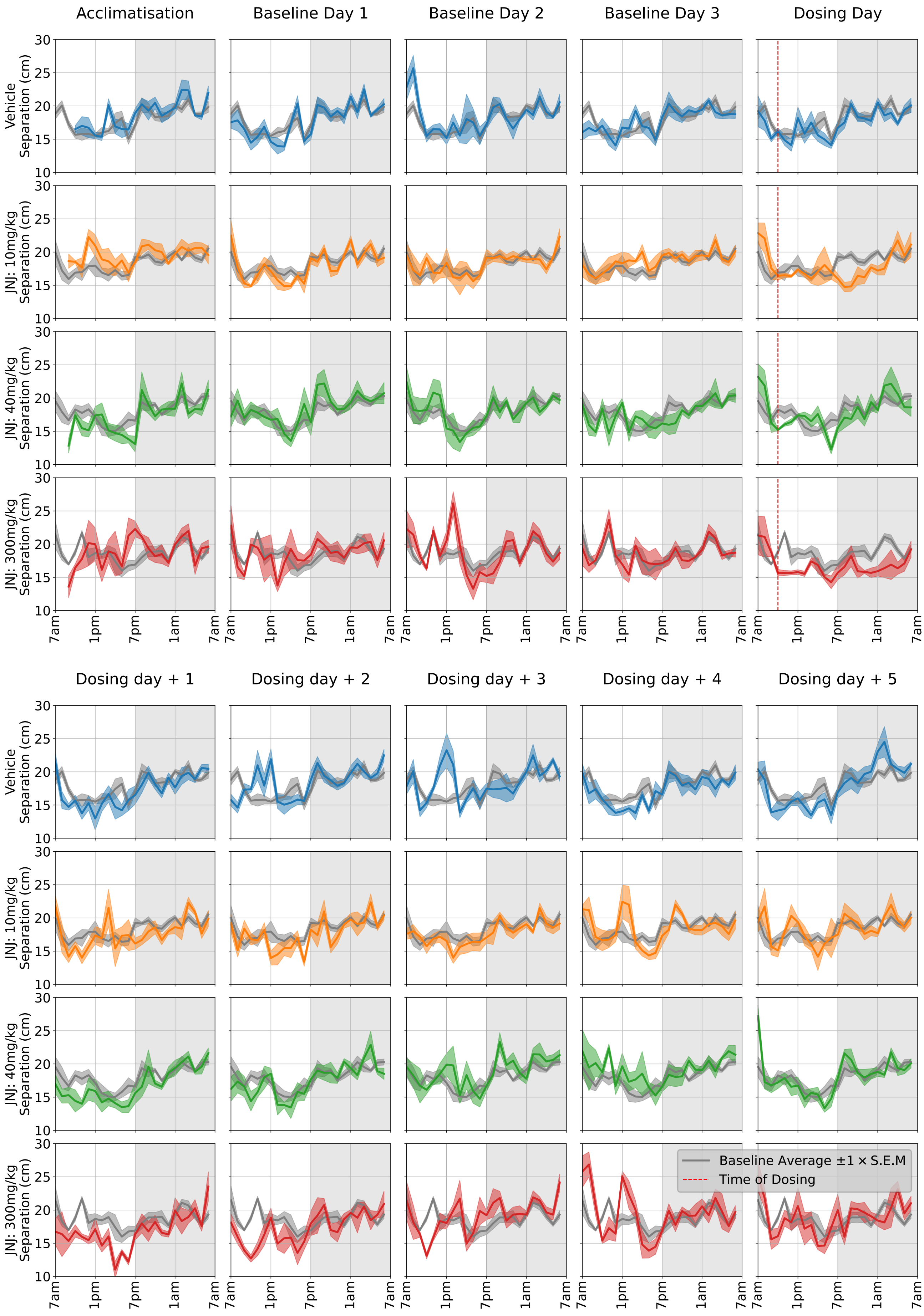

Supplementary Data: JNJ – Drinking (s / min)

Average 24-hour traces (mean ± S.E.M) for each day of the study:

- Sample size is n=6 for duration of recording.

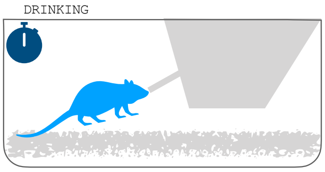

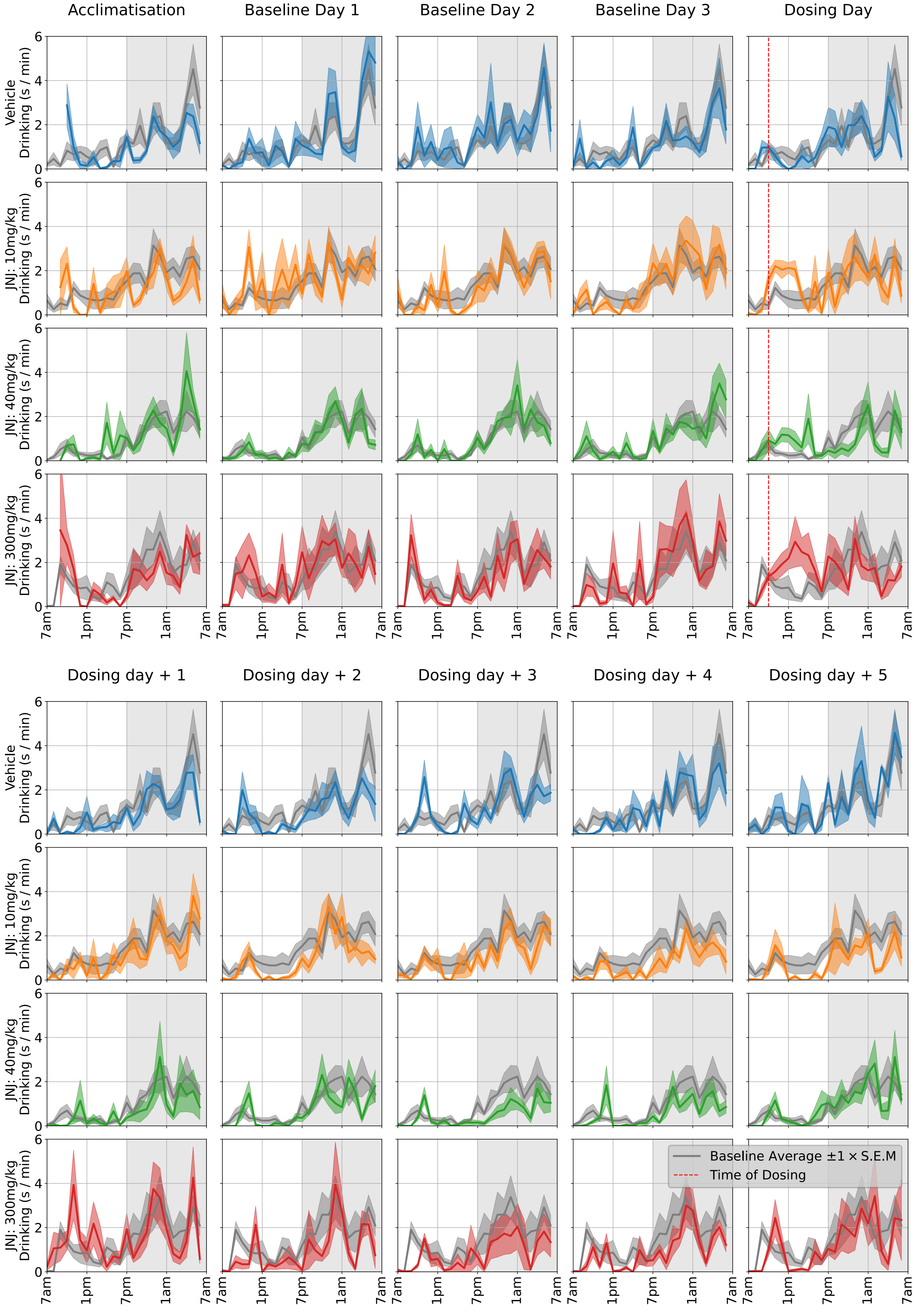

Supplement: Supplementary file 2 [file Supplementaryfile1.pdf]
